# RNA-Seq Analysis of the Expression of Genes Encoding Cell Wall Degrading Enzymes during Infection of Lupin (*Lupinus angustifolius*) by *Phytophthora parasitica*

**DOI:** 10.1371/journal.pone.0136899

**Published:** 2015-09-02

**Authors:** Leila M. Blackman, Darren P. Cullerne, Pernelyn Torreña, Jen Taylor, Adrienne R. Hardham

**Affiliations:** 1 Plant Science Division, Research School of Biology, College of Medicine, Biology and Environment, The Australian National University, Canberra ACT, Australia; 2 Agriculture Flagship, CSIRO, Canberra ACT, Australia; Agriculture and Agri-Food Canada, CANADA

## Abstract

RNA-Seq analysis has shown that over 60% (12,962) of the predicted transcripts in the *Phytophthora parasitica* genome are expressed during the first 60 h of lupin root infection. The infection transcriptomes included 278 of the 431 genes encoding *P*. *parasitica* cell wall degrading enzymes. The transcriptome data provide strong evidence of global transcriptional cascades of genes whose encoded proteins target the main categories of plant cell wall components. A major cohort of pectinases is predominantly expressed early but as infection progresses, the transcriptome becomes increasingly dominated by transcripts encoding cellulases, hemicellulases, β-1,3-glucanases and glycoproteins. The most highly expressed *P*. *parasitica* carbohydrate active enzyme gene contains two CBM1 cellulose binding modules and no catalytic domains. The top 200 differentially expressed genes include β-1,4-glucosidases, β-1,4-glucanases, β-1,4-galactanases, a β-1,3-glucanase, an α-1,4-polygalacturonase, a pectin deacetylase and a pectin methylesterase. Detailed analysis of gene expression profiles provides clues as to the order in which linkages within the complex carbohydrates may come under attack. The gene expression profiles suggest that (i) demethylation of pectic homogalacturonan occurs before its deacetylation; (ii) cleavage of the backbone of pectic rhamnogalacturonan I precedes digestion of its side chains; (iii) early attack on cellulose microfibrils by non-catalytic cellulose-binding proteins and enzymes with auxiliary activities may facilitate subsequent attack by glycosyl hydrolases and enzymes containing CBM1 cellulose-binding modules; (iv) terminal hemicellulose backbone residues are targeted after extensive internal backbone cleavage has occurred; and (v) the carbohydrate chains on glycoproteins are degraded late in infection. A notable feature of the *P*. *parasitica* infection transcriptome is the high level of transcription of genes encoding enzymes that degrade β-1,3-glucanases during middle and late stages of infection. The results suggest that high levels of β-1,3-glucanases may effectively degrade callose as it is produced by the plant during the defence response.

## Introduction

A plant’s first main line of defence against invading pathogens is the plant cell wall. Plant cell walls are composed of an intricate network of complex polysaccharides and glycoproteins, often encased in aromatic polymers such as lignin [1,2]. Within the wall, cellulose microfibrils formed from unbranched chains of β-1,4-glucans provide a scaffold with high tensile strength; hemicellulose molecules crosslink cellulose microfibrils and pectins form a matrix within which wall components are embedded [2]. Hemicelluloses, defined by their solubility at alkaline pH, have a backbone chain of β-1,4-linked glucose, xylose or mannose residues to which side chains containing arabinose, xylose, galactose and fucose residues are attached. Pectins contain a high proportion of α-1,4-linked galacturonic acid residues. The most abundant pectin is homogalacturonan, an unbranched chain of α-1,4-galacturonic acid residues. Other pectins include rhamnogalacturonan I (RGI) and rhamnogalacturonan II (RGII). RGI has a backbone of the repeating disaccharide α-1,4-galacturonic acid-α-1,2-rhamnose to which arabinan and arabinogalactan-based side chains are attached. RGII is a highly branched and complex dimeric polysaccharide which appears to be highly resistant to degradation [1,3–5]. Residues in hemicelluloses, homogalacturonan and RGI may be modified by methyl or acetyl esterification [6]. Digestion of the plant cell wall components by pathogens can release oligosaccharide fragments that trigger plant defence responses including wall strengthening through additional crosslinking of wall components and deposition of β-1,3-glucans (callose), polyphenols and other compounds [7–9].

In order to invade and colonise plants, pathogens secrete a diverse range of cell wall degrading enzymes (CWDEs) that target pectins, hemicelluloses, cellulose, β-1,3-glucans and glycoproteins [10]. Despite the failure of early attempts (e. g. [11]), more recent studies have demonstrated the essential role pathogen CWDEs play in plant infection [12–18]. To aid identification and functional characterization of CWDEs and other carbohydrate-active enzymes (CAZymes), protein motifs associated with specific catalytic activities have been classified into sequence-related CAZyme families [19]. There are six classes of CAZyme modules, namely glycosyl hydrolases (GHs), polysaccharide lyases (PLs), carbohydrate esterases (CEs), auxiliary activities (AAs), carbohydrate-binding modules (CBMs) and glycosyltransferases (GTs). Proteins containing modules from the first five groups are involved with carbohydrate degradation. Individual CAZyme proteins may contain more than one CAZyme module.

Why do phytopathogenic fungi and Oomycetes often have large numbers of CWDE genes? Are multiple enzymes that target the same linkage within a carbohydrate required because of subtle differences in the nearby molecular environment or because of a need for differential regulation of their expression, for example, according to substrate availability or pH? What proportion of an organism’s CWDE gene complement is expressed during plant infection? To what extent is the CWDE transcriptional profile influenced by host species or tissue type and how does it change during course of infection? These are some of the important research questions currently being addressed through studies of pathogen transcriptomes. Although transcriptomic studies do not give a direct measure of the levels of active enzymes, they constitute a powerful analytical tool that provides a valuable indication of CWDE production.

Oomycetes are fungus-like organisms that include many important plant pathogens that cause widespread ecological damage and agricultural losses [20–25]. The genomes of over 30 Oomycetes, including those with biotrophic, necrotrophic or hemibiotrophic lifestyles, have now been sequenced and, in a number of cases, their complements of CWDEs determined [26–29]. The genome of *Phytophthora parasitica*, the Oomycete pathogen central to the current study, contains 431 CWDEs that have modules from 34 GH, three PL, eight CE, four AA and 15 CBM CAZyme families [26]. Some information on patterns of *P*. *parasitica* CWDE gene expression during both *in vitro* and *in planta* growth has been obtained in expressed sequence tags (ESTs) and microarray studies [30–33] but a comprehensive analysis of the *P*. *parasitica* infection transcriptome is still lacking. To address the gap in our understanding of controls of CWDE gene transcription and the functional importance of CWDEs in establishing plant disease, we have used RNA-Seq analysis to determine *P*. *parasitica* CWDE transcriptional profiles during a 60-h time-course of infection of young lupin roots, a model plant-*Phytophthora* pathosystem we have developed. Our results reveal that CWDE genes are amongst those that are the most highly upregulated during lupin root infection. Using our prior detailed characterisation of the CWDE gene complement in *P*. *parasitica* [26], we have been able to show that not only are there global cascades of expression of CWDE genes directed towards the major classes of plant cell wall polysaccharides, but there are also intra-substrate expression cascades of genes encoding enzymes that attack particular polysaccharides. A general principle to emerge is that polysaccharide backbones come under attack before terminal residues, disaccharides or side chains. Our transcriptome results highlight high levels of expression of cellulose-directed CBM1 and β-1,3-glucanase genes during the late phase of infection. CBM1 enzymes could target and dismantle the cellulosic framework within the cell wall. β-1,3-glucanases could neutralize one of the central features of the plant basal defence response namely, callose deposition in cell wall appositions (papillae).

## Materials and Methods

### P. parasitica-lupin (Lupinus angusifolius var. Gungurru) infection assay

*P*. *parasitica* H1111 (ATCC MYA-141), originally isolated by Dr David I. Guest (University of Sydney), was cultured and zoospores released as described in Robold and Hardham [34]. This isolate was collected on the same occasion and at the same location as the isolate of *P*. *parasitica* used as the primary isolate in the *P*. *parasitica* INRA-310 Sequencing Project (http://www.broadinstitute.org/). Lupin seeds were surface sterilized and germinated in moist vermiculite at 23°C in the dark. Germinated seedlings were washed and those with roots 25–30 mm in length were suspended over 200 ml of water (for the mock inoculation) or *P*. *parasitica* zoospore suspension at 1,000 zoospores per ml such that the apical 20 mm of the roots was immersed in the liquid. After 10 minutes, the seedlings were transferred to 150 x 15mm Petri dishes lined with moist filter paper and secured under a wooden applicator stick. The Petri dishes were sealed, placed in a vertical position and incubated at 23°C. Inoculated roots were sampled at 12, 18, 24, 30, 36, 42, 48, 54 and 60 hours post-inoculation (hpi). At each time point, 24 roots (four biological replicates with six roots in each) were excised at the hypocotyl, weighed, frozen in liquid N_2_ and stored at -80°C. Mock inoculated roots were collected at 0 h.

### Quantitation of lupin root infection

Inoculated and uninoculated root samples were ground in liquid nitrogen and a small portion of the powder removed for extraction of genomic DNA (gDNA) by the method outlined in [35]. The relative amounts of *P*. *parasitica* and lupin gDNA were determined using quantitative real-time PCR (qPCR) on a Rotor-Gene 3000 Real Time Thermal Cycler with primers specific for the *P*. *parasitica* gene *WS041* (PPTG_09948) or the lupin nitrilase 4A gene (*NIT4A*; NCBI accession number DQ241759). Primer sequences are listed in S1 Table. qPCR runs were conducted using four technical replicates for each sample, with approximately 300 ng gDNA, 150 nM primers and FastStart SYBR Green Master Mix (Roche Applied Science, Mannheim, Germany). Amplification settings were an initial step of 95°C for 6 min followed by 36 cycles of 15 s at 95°C, 20 s at 60°C and 30 s at 72°C, with data acquired at the end of each annealing step. Data were analysed using the comparative quantification function of the RotorGene v2.0.3 software (Qiagen Pty Ltd, Hilden, Germany).

### RNA isolation and transcriptome sequencing

Total RNA was extracted with a Qiagen RNeasy Midi Kit using shredder columns from a Qiagen Plant RNeasy Kit following the manufacturer’s instructions. gDNA was removed by on-column digestion with DNase (Qiagen) at twice the concentration recommended by the manufacturer. The concentration of total RNA was determined by spectrometry and confirmed by running approximately 1 μg on an RNase-free 1.5% agarose gel in TAE (40 mM Tris, 1 mM EDTA, 20 mM acetic acid) and staining with Red Safe nucleic acid stain (iNtRON Biotechnology, Kyunggi-do, Korea). Three of the four biological replicates for each time point were selected for transcriptome sequencing after considering their ratio of *P*. *parasitica*:lupin gDNA (hereafter referred to as the pathogen load), and the quality and concentration of their total RNA.

RNA library preparation and sequencing were conducted at the Australian Genome Research Facility (Parkville, Victoria). The three biological replicates for each time point were multiplexed and run in a single Illumina HiSeq 2000 lane, generating 50-bp single-end reads using the Illumina Consensus Assessment of Sequence and Variation (CASAVA) pipeline (v1.8.2). Sequence quality was assessed using FastQC (version 0.10.1, http://www.bioinformatics.babraham.ac.uk/projects/fastqc/). Quality scores for all samples were >30 (S2 Table) and no trimming was required. The RNA-Seq data have been deposited in the NCBI Sequence (Short) Read Archive with the SRA accession number SRP061812.

### RNA-Seq data analysis

Reads from all samples, including the mock-inoculated roots, were aligned to version 2 of the *P*. *parasitica* genome (phytophthora_parasitica_inra-310_2_supercontigs.fasta) using BioKanga (http://code.google.com/p/biokanga/; v 1.12.1) with default parameters, which includes exhaustive search and allowance for five mismatches across the 50-bp reads. BioKanga has been deliberately developed for non-model genome analysis and incorporates extensive optimisation to deal with increased uncertainty when aligning to non-reference genomes. Reads that mapped to only a single location were placed in a “unique location” data set and used to analyze expression patterns of individual genes. In a “multiple location” data set, reads that mapped to more than one location were retained after random assignment to one position. In both cases, reads that aligned to regions delimitated by the coordinates of the *P*. *parasitica* predicted transcripts, which exclude 5’ and 3’ untranslated regions and introns, gave rise to the raw count values. Some reads mapped to transcripts that had splice variants (designated T0, T1 and T2 in the *P*. *parasitica* genome project data). In these cases, the T0 transcript variant was used.

The DESeq approach [36] and associated R program (http://bioconductor.org/packages/release/bioc/html/DESeq.html) was used to normalise the mapped reads across the unique or multiple location data sets, estimate dispersion or variance for each gene using the replicates within the experimental design and determine statistical support for differential expression. Normalised reads per kilobase of predicted transcript (NRPK) were then calculated. The effectiveness of the normalisation algorithm in adjusting for pathogen load was assessed by examining the NRPK values for five *P*. *parasitica* genes shown by qPCR to be constitutively expressed under a range of conditions [37]. These genes were peptidyl prolyl isomerase 2 (PPTG_02092), phospholipase A2 (PPTG_08636), 40S ribosomal protein S3A (*WS021*, PPTG_07764), ubiquitin-conjugating enzyme (PPTG_08273), and *WS041*.

Analysis of the NRPK RNA-Seq data was based on the median value of the three biological replicates for each time point. In consideration of the digital nature of RNASeq, a measure of the overall level of transcription of each CWDE gene was obtained by summing the median NRPK values for each of the 30–60 hpi time points. Genes were deemed to be expressed if the median of the raw counts at any time point was ≥20 and the total of the median NRPK values across the time-course was ≥5. In three cases, the annotation of the *P*. *parasitica* genome incorrectly split genes into two transcripts [26]. For these transcripts, the NRPKs of the two transcripts have been added together.

For each transcript, differential expression between time points was assessed by transforming the normalised unique location data set to log base 2 (log2) and calculating the difference between the highest and lowest median, non-zero log2 values. Because only non-zero values were included, this provided a conservative measure of fold changes. It is generally accepted that genes are differentially expressed if their log2 value is ≥ 2 [38,39].

The pattern of expression of CWDE families was determined using NRPK values from the multiple location data set. Gene expression profiles were categorised as early (≤36 hpi), middle (42 and 48 hpi) or late (54 and 60 hpi) according to the time at which transcript levels peaked. At each time point, the NRPKs of transcripts for proteins targeting a predicted substrate were summed to give an indication of the total transcript level for each CWDE family. Genes encoding CAZyme proteins that are not involved in the degradation of cell wall polysaccharides or glycoproteins were not included in the current analysis although the raw data for these genes can be found in S3 Table. They included amylase, invertase and trehalase. Genes with a total NRPK value across the time-course of <50 were not included in the detailed analyses of CAZyme expression patterns.

### qPCR analysis of selected genes

cDNA was prepared from the same RNA samples used for RNA-Seq analysis, and qPCR conducted with three or four technical replicates using Quantifast SYBR Green (Qiagen), as described in Blackman and Hardham [40]. Gene transcript levels in each sample were calculated relative to their value in 3-h germinated cyst cDNA [41] using the RotorGene comparative quantification function. Relative expression levels were calculated with respect to levels of expression of the constitutively-expressed, normalising gene, *WS041* in each sample.

## Results

### *P*. *parasitica*-lupin infection assay

**Fig 1:**
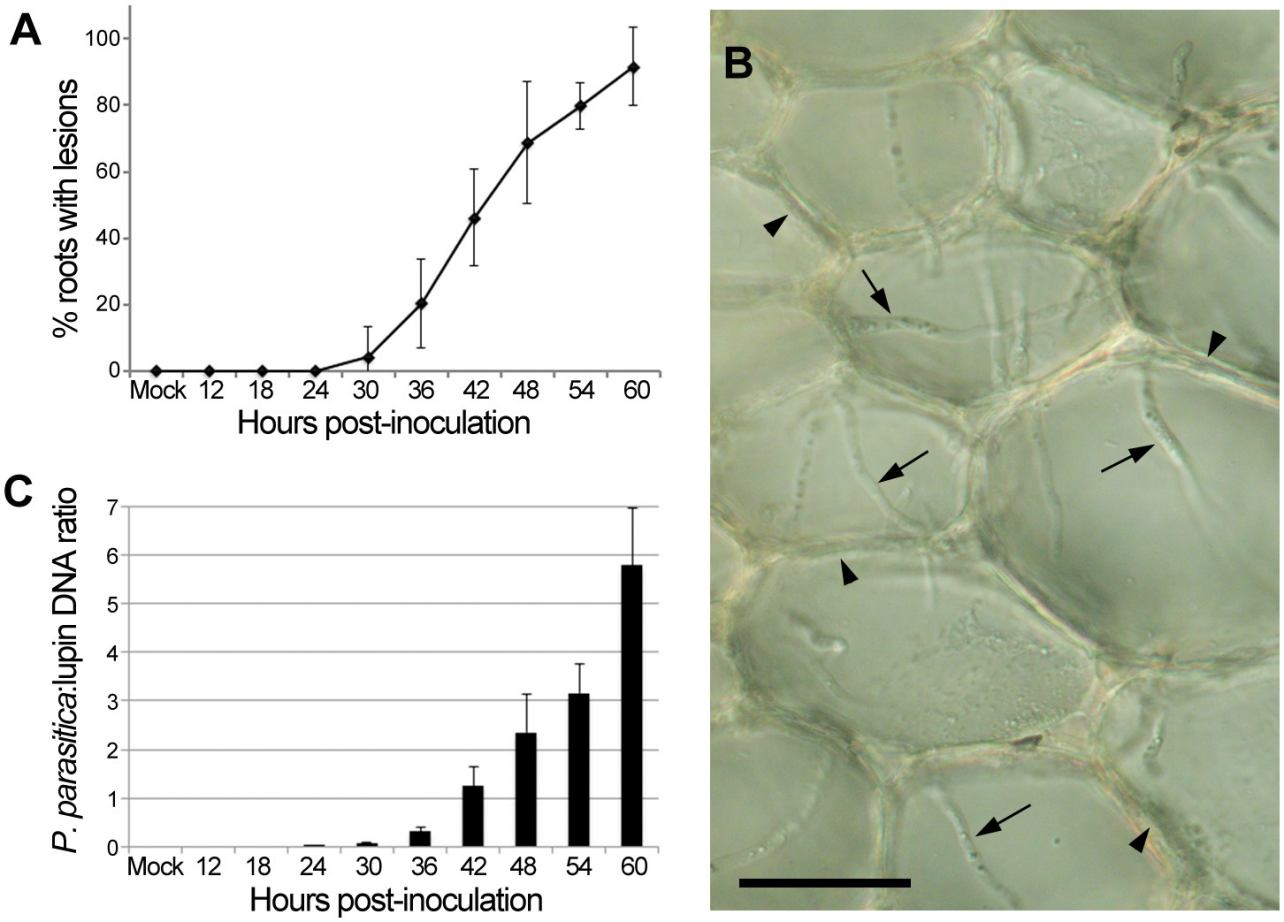
Infection of lupin roots by *P*. *parasitica*. **(A)** Percentage of roots with visible lesions during the infection time course, 0 hpi to 60 hpi. (n = 12). **(B)** Transverse hand section of a lupin root inoculated with *P*. *parasitica* zoospores. By 27 hpi, *P*. *parasitica* hyphae grow both intercellularly (arrowheads) and intracellularly (arrows) during colonisation of the root cortex. Scale bar = 50 μm. **(C)** Ratio of *P*. *parasitica* DNA:lupin DNA as determined by qPCR measurement of the levels of the *P*. *parasitica WS041* gene and the lupin nitrilase gene 4A (*NIT4A*). Data are from the three biological replicates chosen for RNA-Seq transcriptome analysis. *P*. *parasitica* DNA was not detected in mock-inoculated or 12 hpi or 18 hpi samples. Error bars indicate the standard error of the mean.

Visible lesions began to develop on the lupin roots about 30 hpi, close to the site that had been at the surface of the zoospore suspension during inoculation (Fig 1A). Because *Phytophthora* zoospores are negatively geotactic, they concentrate at the surface of the liquid. This means that the density of zoospores in the rhizosphere will increase over the 10-min inoculation period, and the local zoospore concentration will be greater than the overall value of 1,000 zoospores/ml within the 200 ml of inoculation liquid. By 60 hpi, most of the length of the root was necrotic. Encysted spores on the root surface germinated 30–90 min after inoculation and the germ tubes penetrated the root epidermis over the ensuing 1–2 h. Hyphae grew intercellularly or intracellularly through the root cortex (Fig 1B) and reached the centre of the root by 24 hpi.

The ratio of *P*. *parasitica* to lupin gDNA in the four biological replicates of the mock-inoculated and inoculated lupin roots was determined by qPCR using primers specific for *P*. *parasitica WS041* and *L*. *angustifolius NIT4A* genes. No *P*. *parasitica* DNA was detected in the mock-inoculated, or in the 12 hpi and 18 hpi samples (Fig 1C). The ratio of *P*. *parasitic*a gDNA to lupin gDNA increased from 0.02 at 24 hpi to 5.80 at 60 hpi. Three of the four biological replicates with the most similar *P*. *parasitica*:lupin DNA ratios at each time point were selected and submitted for RNA-Seq sequencing.

### Methodology for RNA-Seq data analysis

Approximately 60 million, 50-bp, single-end reads were obtained per sample (S2 Table). The RNA-Seq data (S3 Table) included reads that mapped to 95 *P*. *parasitica* transcripts in the mock-inoculated samples. These included genes encoding highly conserved proteins such as actin, tubulin, elongation factor-1 alpha, calmodulin, peptidyl-prolyl cis-trans isomerase, ribosomal proteins, and heat shock proteins 70 and 90. These 95 genes were eliminated from further consideration in the RNA-Seq analysis leaving 20,403 predicted transcripts. Only one of the 95 was predicted to encode a CWDE (a GH28 polygalacturonase, PPTG_15164) and its expression was examined using qPCR and *P*. *parasitica* primers that did not cross-react with the lupin orthologue. qPCR assays failed to detect *P*. *parasitica* gDNA in the 12 hpi and 18 hpi samples (Fig 1C), and the numbers of reads mapped to *P*. *parasitica* transcripts in the 12 hpi, 18 hpi and 24 hpi samples were too low for analysis (S2 Table). Thus the RNA-Seq data from these early time points were not included in the analysis of pathogen gene expression. When an NRPK value at 30 hpi was high, gene expression was analysed by qPCR in 24 hpi and later samples.

To check that the DESeq normalisation algorithm produced effective scaling for pathogen load, the NRPK values of five housekeeping genes (see Materials and Methods section) were examined. Transcript levels of these five genes covered a range of two orders of magnitude, but after application of the scaling algorithm, variation in their NRPK values during the infection time-course was only 1.25–1.6 fold (S1 Fig). This indicated that the scaling algorithm made appropriate adjustments for differences in pathogen load.

### *P*. *parasitica* genes most highly or differentially expressed during lupin root infection

The overall level of expression of each *P*. *parasitica* transcript during the 30–60 hpi time-course was estimated by summing the median NRPK values from each time point in the unique location data set. Of the 20,403 predicted *P*. *parasitica* transcripts, 12,962 were expressed according to the criteria that the median raw read count for the gene was ≥20 at at least one time point and that the total NRPK value across the time-course was ≥5. The infection transcriptomes included 278 of the 431 known *P*. *parasitica* CWDEs (S3 Table). The top 200 most highly expressed genes included four CWDEs, namely GH5/CBM43: PPTG_01939, GH81: PPTG_13594, GH17: PPTG_17187 and CBM1: PPTG_06045 (S4 Table). The latter gene contains two CBM1 domains and no other CAZyme modules.

Genes were deemed to be differentially expressed during the time-course if comparisons of normalised read counts that were ≥1 had a log2 ratio ≥2, indicative of a four-fold or more change in expression. Application of these criteria revealed that 5,406 *P*. *parasitica* genes were differentially expressed between 30 and 60 hpi. The most highly differentially expressed gene encodes an elicitin-like protein (PPTG_15237) that was ranked fifth in terms of transcript level and that had a >500-fold increase in expression between 30 and 54 hpi. The top 200 differentially expressed genes included 21 CWDEs (Table 1 and S5 Table).

**Table 1:** CWDEs in the top 200 differentially expressed genes between 30 hpi and 60 hpi.

Rank	Log2 ratio	CAZyme family	Transcript No.
13	7.81	CBM1: contains a cellulose-binding domain (Rank 87 in top 200 for transcript abundance)	PPTG_06045
28	7.02	GH1: β-1,4-glucosidase	PPTG_12009
33	6.93	GH5_20: endo-β-1,4-glucanase	PPTG_03846
46	6.56	GH30: β-glucosidase	PPTG_09216
53	6.44	CBM1: contains a cellulose-binding domain	PPTG_05833
54	6.43	GH53: endo-β-1,4-galactanase	PPTG_19167
64	6.27	GH28: polygalacturonase	PPTG_15171
82	5.96	GH16: endo-β-1,4-glucanase acting on hemicellulose	PPTG_12471
93	5.82	GH1: β-1,4-glucosidase	PPTG_12010
100	5.77	GH17: β-1,3-glucanase	PPTG_11267
102	5.74	CBM1: contains a cellulose-binding domain	PPTG_13482
114	5.68	CE13: pectin acetylesterase	PPTG_10125
127	5.54	GH105: unsaturated rhamnogalacturonyl hydrolase	PPTG_07904
129	5.54	CE8: pectin methyl esterase	PPTG_09705
150	5.38	GH1: β-1,4-glucosidase	PPTG_05794
153	5.36	GH53: endo-β-1,4-galactanase	PPTG_19168
174	5.26	CBM1: contains a cellulose-binding domain	PPTG_04643
183	5.24	CBM1: contains a cellulose-binding domain	PPTG_17441
188	5.21	GH12: β-1,4-glucanase	PPTG_19378
190	5.21	GH7: β-1,4-glucanase	PPTG_07017
195	5.20	GH1: β-glucosidase	PPTG_18270

The rank, log2 ratio, CAZyme family, predicted function, and transcript numbers are shown.

### Global patterns of CWDE gene expression

**Fig 2:**
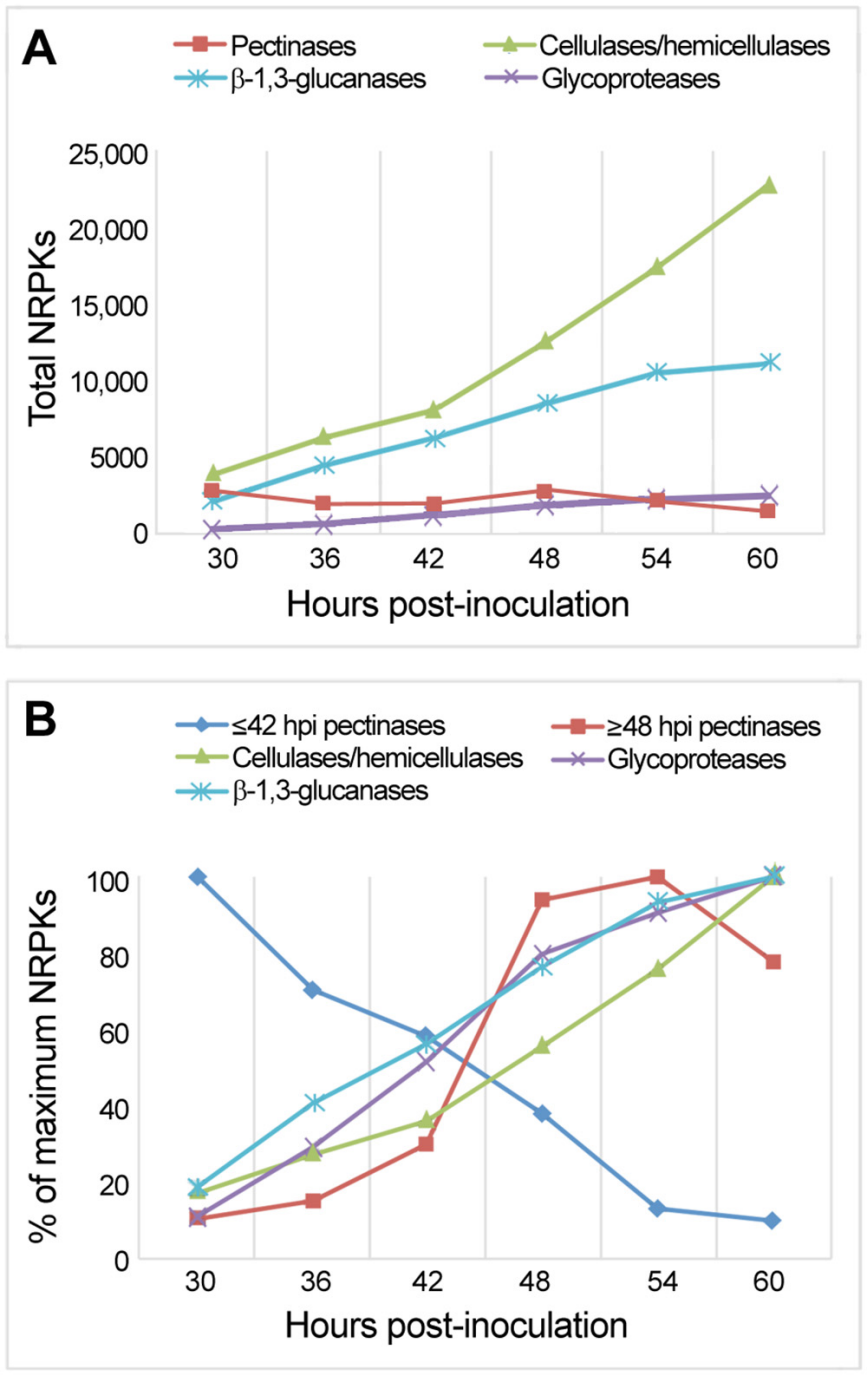
Total NRPKs from the multiple location data set for genes targeting major categories of wall components. Because many genes act on cellulose and hemicelluloses, counts for these two substrates have been combined. **(A)** Total NRPK counts show the relative transcript abundance for each substrate category. **(B)** Total NRPK counts expressed as a percentage of the maximum counts show trends in the expression profiles over time. Genes whose products target pectins have been assigned to the first and second halves of the 60-h infection time-course.

Evaluation of global patterns of expression of genes targeting the major categories of wall components, namely pectins, cellulose, hemicelluloses, β-1,3-glucans and glycoproteins used the multiple location data set. In terms of total transcript abundance, there are similar levels of pectinases and cellulases/hemicellulases at 30 hpi and about half that number of β-1,3-glucanases (Fig 2A). As infection proceeded, the CWDE transcriptome became dominated by cellulases/hemicellulases and β-1,3-glucanases. Calculation of the total NRPK values as a percentage of the maximum transcript number for each category of enzymes, revealed that a cohort of pectinases were expressed most strongly during the first half of the infection time-course (Fig 2B). This was not the case for enzymes targeting the other substrate categories. As transcript levels of the early cohort of pectinases decreased, transcript levels for the other pectinases and for enzymes targetting cellulose, hemicellulose, β-1,3-glucans and glycoproteins increased.

**Fig 3:**
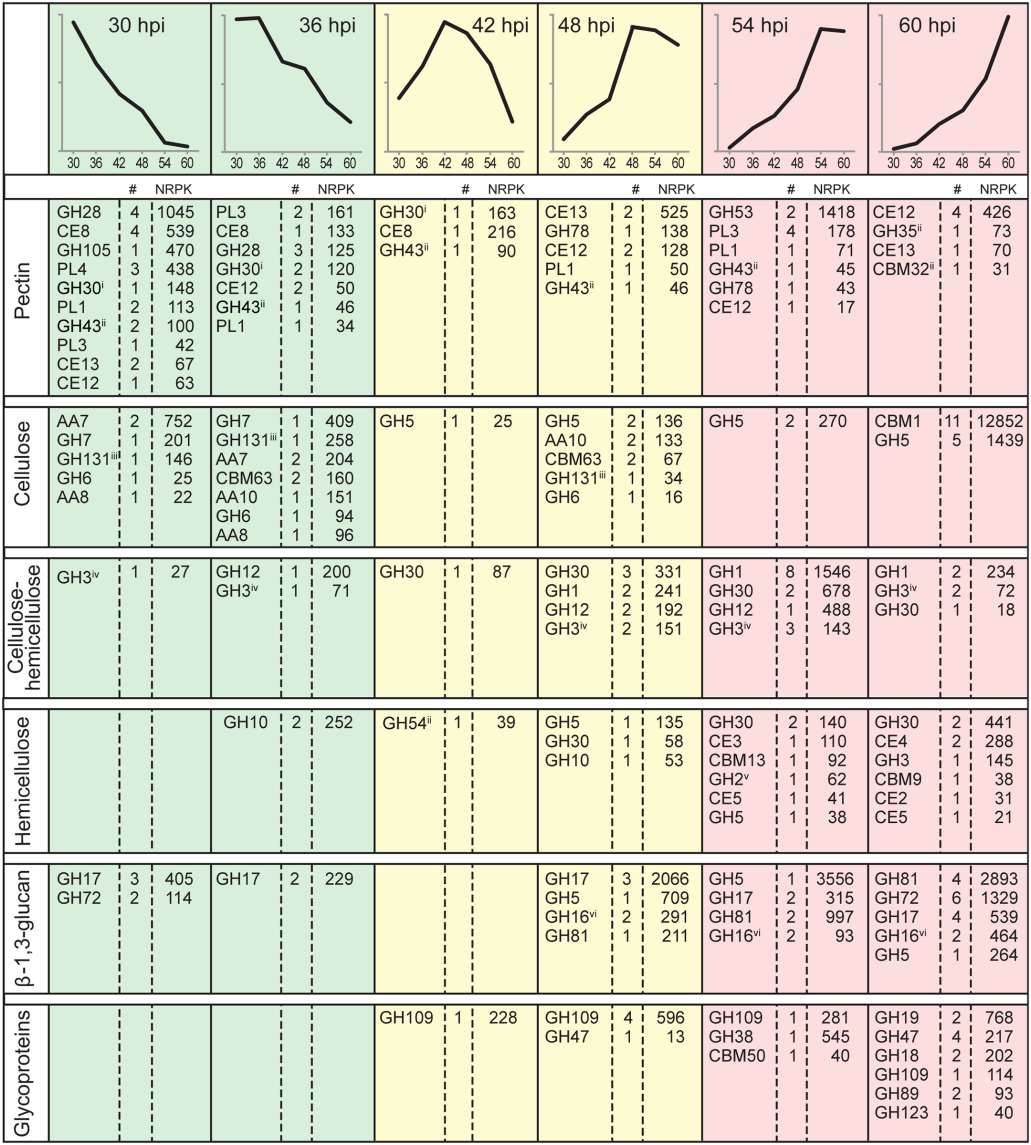
Summary of expression profiles of *P*. *parasitica* CWDEs during the infection of lupin roots. The top row shows representations of the six main expression profiles displayed by *P*. *parasitica* CWDEs. CAZyme families have been assigned to putative polysaccharide category according to homology with characterised CAZymes. Within each major wall polysaccharide category, the NRPK values of CAZyme family members whose expression peaks at the same time (the number of genes is shown in the column labelled #) have been summed and the total peak NRPK value shown in the columns labelled NRPK. CAZyme families whose members act on multiple substrates are indicated by superscripts. These CAZyme families have be allocated to a selected substrate according to recent literature on their most likely function. The superscripts indicate their possible targets: ^i^pectin and glycoproteins; ^ii^pectin, hemicellulose and glycoproteins; ^iii^cellulose and β-1,3-glucans; ^iv^cellulose, hemicellulose and glycoproteins; ^v^hemicellulose and glycoproteins; ^vi^hemicellulose and β-1,3-glucans.

In order to assess the contribution of CAZyme families and individual genes to the transcript pool at each time point, median NRPK values from the multiple data set were used to determine the time at which transcript abundance peaked for each gene. These NRPK values were then summed for all members of the CAZyme family whose expression peaked at that time. The results are summarised in Fig 3 which shows the number of genes in each CAZyme family and the total NRPK values for the cohort of genes whose peak of expression occurred at that time, grouped according to putative substrate targeted. The table provides an overview of the CAZyme expression data underlying the global trends displayed in Fig 2. At a glance, the predominance of pectinase expression early in infection and of CBM1 and β-1,3-glucanase expression late in infection is evident. The stylised models along the top of Fig 3 are based on the RNA-Seq data presented in S2–S7 Figs Together with Fig 3 and S6 Table, S2–S7 Figs present the core results of our study in terms of CWDE gene expression profiles during early (30–36 hpi), middle (42–48 hpi) and late (54–60 hpi) infection, grouped according to potential substrates and targeted linkages.

### Pectin degradation

The *P*. *parasitica* genome contains 108 CWDEs that are likely to act specifically on pectins, and another 15 that may also act on hemicellulose and glycoproteins [26]. These 123 CWDEs come from 14 CAZyme families. During the 60-h lupin root infection time-course, 76 of the 123 genes were expressed, including at least one representative from each of the 14 CAZyme families (S6 Table). A total of 43, 21 and 12 genes from 10, seven and four CAZyme families were predominantly expressed during early (30–36 hpi) middle and late infection respectively (S2 Fig and S6 Table).

#### Homogalacturonan

Homogalacturonan is a polymer of α-1,4-linked galacturonic acid residues (S2 Fig). GH28 endopolygalacturonases cleave the α-1,4-linkage between galacturonic acid residues. Two of the eight *P*. *parasitica* GH28 genes had transcript levels nearly 10-fold higher than the other six GH28 genes (S6 Table). RNA-Seq and qPCR data showed that the highest transcript levels for these two genes occurred at 24–30 hpi. PL1 and PL3 enzymes degrade the α-1,4-linkage between galacturonic acid residues at the non-reducing end of homogalacturonan, with a preference for unesterified residues (pectate lyase activity). PL1 also acts on terminal unesterified residues at the reducing end (pectate lyase activity) and on methyl esterified residues (pectin lyase activity). Differential expression and transcript levels of PL1 and PL3 genes were generally low (Fig 2, S6 Table and S2 Fig) but both RNA-Seq and qPCR data showed that two of the most highly expressed PL1 genes (PPTG_12896 and PPTG_17499) and the two most highly expressed PL3 genes were preferentially expressed early.

There are no side chains on the homogalacturonan backbone but methyl and acetyl groups may be attached to galacturonic acid residues (S2 Fig). CE8 pectin methyl esterases remove the methyl groups. All P. *parasitica* CE8 genes were predominantly expressed at ≤ 36 hpi (qPCR and RNA-Seq data; Fig 3, S6 Table and S2 Fig). Two of the nine CE8 genes (PPTG_06239 and PPTG_10338) were in the top 20% of CWDEs in terms of overall transcript levels.

#### Rhamnogalacturonan

The backbone of RGI is attacked by proteins in the GH105, GH78, PL4 and CE12 families (S2 Fig). GH105 enzymes target the α-1,2-linkage between galacturonic acid and rhamnose residues, GH78 enzymes act on the terminal non-reducing end of the RGI backbone and PL4 enzymes target the α-1,4-linkages between rhamnose and galacturonic acid. The single *P*. *parasitica* GH105 and the three PL4 genes were all most highly expressed at 30 hpi (Fig 3, S6 Table and S2 Fig).

Proteins in GH30, GH35, GH43 and GH53 CAZyme families target the side chains of RGI polysaccharides (S2 Fig). The GH30 family is functionally diverse and includes some members that target cellulose or hemicellulose (http://www.cazy.org). Our in-depth sequence analysis suggests that four GH30 proteins are likely to have endo-β-1,6-galactanase activity and act on RGI and AGP side chains [26]. GH43 proteins primarily act on internal and terminal β-1,3-linked galactose and terminal α-1,3- and α-1,5-linked arabinose residues found in RGI, AGPs and hemicellulose side chains. The RNA-Seq and qPCR data indicated that during lupin root infection, genes from GH30 and GH43 families were mainly expressed during early and mid-infection. One of the four GH30 had overall transcript levels that placed it in the top 20% of the CWDEs in terms of expression level.

The cohort of putative RGI-directed transcripts late in infection was dominated by GH53 genes (S6 Table and S2 Fig). GH53 enzymes act on the β-1,4-linkage between galactose residues in the side chains of RGI. One GH53 gene (PPTG_19167) was the seventh most highly expressed *P*. *parasitica* CWDE gene and had a >64-fold increase in expression between 36 hpi and 48 hpi (S6 Table). The single GH35 protein in *P*. *parasitica* is predicted to be a β-galactosidase acting on terminal β-galactose residues in hemicellulose and RGI and terminal β-1,3- and β-1,6-galactans in AGPs [26]. Transcripts for the GH35 gene were relatively low but showed a steady increase during the 60-h time-course of lupin root infection (S6 Table and S2 Fig).

#### CE12 and CE13 pectin acetyl esterases

CE12 and CE13 proteins are pectin acetyl esterases that remove the acetyl group from residues in homogalacturonan and RGI backbones. The 11 CE12 genes expressed during lupin root infection showed the greatest diversity in expression pattern of all the *P*. *parasitica* CAZyme families. Transcript levels for at least one CE12 gene peaked at every time point except 42 hpi (S6 Table and S2 Fig). Of the two CE12 genes in the top 22% of *P*. *parasitica* CWDEs in terms of overall transcript levels, one peaked mid-infection and the other late-infection. CE12 gene expression patterns did not correlate with protein sequence homologies. Two *P*. *parasitica* CE13 acetyl esterases were also in the top 15% of CWDEs in terms of overall transcript levels. One of these CE13 genes peaked during middle infection, with a >50-fold increase in expression between 36 hpi and 48 hpi. The other highly expressed CE13 peaked during late infection (Fig 3, S6 Table and S2 Fig).

### Degradation of cellulose and hemicellulose

Twenty eight *P*. *parasitica* CWDE families have the potential to act on cellulose, hemicellulose or both polysaccharides [26]. There are 217 genes in these 28 families in the *P*. *parasitica* genome and 150 of them were expressed during lupin root infection. Detailed sequence analysis suggests that 34 of the 150 encoded proteins may target other carbohydrates. The expression of the 116 CWDEs that are likely to act on cellulose and/or hemicellulose is discussed in this section.

#### Cellulose

*P*. *parasitica* genes that were expressed and whose encoded proteins potentially act on cellulose came from three AA, four GH and two CBM CAZyme families (Fig 3, S6 Table and S3 Fig). AA7 proteins are oxidases with broad specificity for β-1,4-linked glucans [42]. Transcript levels for the four expressed AA7 genes were highest at 30 hpi and decreased approximately 16-fold by 60 hpi. One AA7 gene (PPTG_19661) was the 14th most highly expressed *P*. *parasitica* CWDE gene during lupin infection. AA8 proteins contain iron reductase domains and may generate reactive oxygen species that could contribute to non-enzymatic degradation of cellulose chains [42]. AA8 genes were also expressed most highly during early infection, although at about 10% the level of AA7 genes. AA10 proteins are secreted oxidases. Transcripts of the most highly expressed AA10 gene peaked at 30 hpi while expression of the other AA10 genes peaked during mid-infection.

GH6 and GH7 proteins have endo-β-1,4-glucanase activity, with GH6 enzymes also releasing disaccharides from the non-reducing end of the glucan chain and GH7 enzymes also releasing disaccharides from the reducing end (S3 Fig). Transcript levels for GH6 and GH7 genes were highest during early infection before decreasing steadily thereafter (Fig 3, S6 Table and S3 Fig). GH7 transcript abundance was about four times higher than that of GH6 genes. GH131 CAZymes are exo-β-1,3/1,6-glucanases with endo-β-1,4-glucanase activity [43]. The highest GH131 transcript levels also occurred during infection (Fig 3, S6 Table and S3 Fig).

GH5 CAZymes have a range of activities and targeted substrates and have been recently divided into 51 subfamilies [44]. Detailed sequence analysis has allowed the *P*. *parasitica* GH5 genes to be separated into three groups with putative activity on (i) β-1,3-glucans, (ii) β-1,4-glucans in cellulose and (iii) β-1,4-mannans in hemicellulose [26]. Expression of the 13 putative GH5 β-1,4-glucanases peaked at 60 hpi (Fig 3, S6 Table and S3 Fig).

Carbohydrate binding domains do not have catalytic activity. CWDEs that contain CBM domains may or may not also contain CAZyme catalytic domains [45,46]. In our analysis of *P*. *parasitica* CWDEs, genes that contained one or more CBM modules and one or more catalytic domains were assigned to the catalytic CAZyme family. Two *P*. *parasitica* CBM families, CBM1 and CBM63, include proteins that are thought to bind to cellulose [26,47] and that lack a catalytic CAZyme domain. The CBM63 genes were, in general, not highly expressed. Transcript levels for the most highly expressed CBM63 gene (66^th^ in overall transcript levels) peaked early in infection (Fig 3 and S6 Table).

In contrast to the generally low expression of CBM63 genes, transcript levels for the majority of the CBM1 genes expressed during lupin root infection was high. Indeed, one CBM1 gene, PPTG_06045, was the most highly expressed *P*. *parasitica* CWDE in the RNA-Seq analysis and three others were also in the top 12 CWDEs. The three most highly expressed CBM1 genes were highly differentially expressed with log2 ratios ≥ 5.7 with their highest expression occurring at 60 hpi (Fig 3, S6 Table and S3 Fig).

#### Cellulose and hemicellulose

Proteins encoded by genes from four *P*. *parasitica* GH families, namely GH1, GH3, GH12 and GH30, have the potential to act on both cellulose and hemicellulose (S4 Fig). All expressed *P*. *parasitica* GH1 proteins potentially target terminal β-1,4-linked glucose residues in both cellulose and hemicellulose. GH3 proteins target the non-reducing end of a range of polysaccharides including β-1,4-glucans of cellulose and hemicellulose, β-1,4-xylans and β-1,4-mannans of hemicellulose, and the terminal α-arabinose residues of AGPs [26]. GH12 proteins act on β-1,4-glucans in cellulose and xyloglucans of hemicellulose. GH30 proteins have a diverse range of activities but 10 of the *P*. *parasitica* GH30 genes that are expressed during lupin root infection have sequence features in common with GH30s from other species that act on the terminal β-1,4-glucose and β-1,4-xylose residues of cellulose and hemicellulose [26,48].

As is evident in Figs 2 and 3, transcript levels for these four cellulose- and/or hemicellulose-targeting families were highest late in infection. Some members of the GH3, GH12 and GH30 families were expressed predominantly during early or middle infection, but transcript levels for these genes were not as high as for those whose expression peaked late in infection (Fig 3, S6 Table and S4 Fig). GH1 dominated the cellulase and/or hemicellulase transcript pool at 54 hpi and 60 hpi. The GH1 PPTG_12102 gene had the 15th highest transcript level of all the *P*. *parasitica* CWDEs and a >32-fold change (log2 ratio >5) in abundance between 30 hpi and 54 hpi. The 15 GH12 genes in the *P*. *parasitica* genome occur in five clusters but there was no evidence of co-regulation of the expression of GH12 genes within a cluster.

#### Hemicellulose

Many proteins encoded by genes in GH, CE and CBM families act on hemicellulose as well as a range of other polysaccharides. In general, only those believed to be specific for hemicellulose are discussed in this section. Although some genes that target hemicellulose were expressed during early or middle infection, the highest transcript levels were seen during late infection (Fig 3, S6 Table and S5 Fig).

GH10 proteins are β-1,4-xylanases that act on xylans, glucuronoxylans and glucuronoarabinoxylans. GH10 was the only family of hemicellulose-directed enzymes whose gene expression peaked during early infection (Fig 3, S6 Table and S5 Fig). Extensive sequence analysis suggests that two GH5 genes (PPTG_03499 and 18368) are likely to encode β-mannosidases and that the GH54 gene PPTG_18202 encodes an α-arabinofuranosidase that acts on terminal α-arabinose in hemicellulose, RGI side chains and AGPs [26,27]. Transcript levels for these three genes were low but peaked during mid-infection.

During late infection, the pool of transcripts encoding enzymes putatively specific for hemicellulose was dominated by GH30, GH30/CBM13 and CE4 genes (Fig 3, S6 Table and S5 Fig). One of the GH30/CBM13 genes was the 16th most highly expressed *P*. *parasitica* CWDE gene. Other families of genes that were expressed late in infection and that are thought to encode hemicellulose-directed enzymes included GH3 β-1,4-xylanases and the CE2, CE3 and CE5 esterases, which remove the acetyl moiety from xylans. CBM9 and CBM13 genes also had expression peaks at 54 hpi or 60 hpi. A GH2 gene thought to encode a mannosidase that acts on terminal β-1,4-mannans in hemicelluloses and N-linked glycoproteins was also expressed late in infection.

### Degradation of β-1,3-glucans

There are 84 genes in the *P*. *parasitica* genome that encode enzymes with the potential to degrade β-1,3-glucans and 53 of these genes were expressed during the infection of the lupin roots (S6 Table). They come from GH5, GH16, GH17, GH81 and GH72 families (Fig 3, S6 Table and S6 Fig). Three GH5 proteins also contain a CBM43 motif and two GH17 proteins also contain a CBM13 domain. As previously acknowledged, there are difficulties in determining CWDE function from bioinformatic analysis and in some cases, there is not yet sufficient information available to make a valid judgement. However, intensive sequence analysis, including proteins with demonstrated enzymatic activity, has provided a strong indication of the likely role of selected GH5 and GH16 in β-1,3-glucan degradation. Because *P*. *parasitica* β-1,3-glucanases could potentially act on *P*. *parasitica* β-1,3-glucans in the pathogen cytoplasm or cell wall, possession of a signal peptide directing secretion, a transmembrane domain (TMD) or a glycosylphosphatidylinositol (GPI) anchor in the putative β-1,3-glucanases is noted in this section.

The *P*. *parasitica* β-1,3-glucanase genes are predominantly expressed 48–60 hpi (Fig 3, S6 Table and S6 Fig). Only GH17 and GH72 families contain genes for which transcript levels were highest early in infection. Evidence available to date indicates that all GH17 and GH72 enzymes are specific for β-1,3-glucans [49–51]. Transcript levels for these genes were low compared to β-1,3-glucanase transcript levels late in infection. One GH17 protein (PPTG_02889) has a TMD and one GH72 protein (PPTG_09844) has a GPI anchor, suggesting that they are likely to be membrane associated [26].

Genes from GH5, GH16, GH17 and GH81 families had peak transcript levels during middle infection. GH5 enzymes may attack a number of different substrates but detailed sequence analysis suggests that the most highly expressed GH5 gene (PPTG_01483) encodes a β-1,3-glucanase genes whose transcription peaked at 48 hpi (S6 Table). GH16 enzymes may attack β-1,3-glucans or xyloglucans [52,53]. For the majority of *P*. *parasitica* GH16 genes, there is no clear indication of their specificity, however, sequence similarities have led to their inclusion as putative as β-1,3-glucanases. The β-1,3-glucanase transcript pool at 48 hpi was dominated by GH17 genes, one of which (PPTG_10954) contains a GPI motif [26]. All seven proteins have classical secretion signals.

The majority of the β-1,3-glucanase genes are most strongly expressed late in infection (Fig 3, S6 Table and S6 Fig). This cohort includes three GH5 genes that contain a CBM43 module, with one gene being the second most highly expressed *P*. *parasitica* CWDE gene. Although GH5 enzymes have a diverse range of substrates, CBM43 modules have been found only in enzymes that target β-1,3-glucans. The cohort also includes genes from GH16, GH17, GH72 and GH81 families, 13 of which are in the top 50 CWDEs in terms of transcript abundance (S6 Table). One GH81 gene is the third most highly expressed CWDE gene. The proteins encoded by all of the late-expressing genes, except for GH72 PPTG_06144, contain either classical or non-classical secretion sequences and 15 contain predicted GPIs or TMDs [26]. Examination of the position of the 16 GH81 genes in the *P*. *parasitica* genome showed that adjacent genes have different expression patterns.

### Glycoprotein degradation

Eighteen CAZyme families in the *P*. *parasitica* genome include genes that encode proteins with the potential to degrade oligosaccharide chains found in *N*- and *O*-linked glycoproteins, arabinogalactan proteins or chitin [26,50,54,55]. In some cases, the families includes proteins with diverse targets but four that are expressed during lupin infection, namely GH18, GH19, GH38, GH47 and GH89, contain a complement of genes whose encoded proteins are thought to be specific for *N*-linked oligosaccharides. Two families, GH109 and GH123, include proteins that can degrade *O*-linked oligosaccharides and five, GH3, GH30, GH35, GH43 and GH54, include proteins that have the potential to target arabinogalactan proteins as well as cellulose, hemicellulose and pectins.

As indicated in Fig 3 and S7 Fig, genes whose encoded proteins target glycoproteins were predominantly expressed late in infection. GH18 and GH19 proteins cleave the β-1,4-linkage between *N*-acetylglucosamine residues at the base of the oligosaccharide chain while GH38 and GH47 proteins act on linkages between terminal mannose residues (S7 Fig). GH19 and GH38 transcripts dominated the cohort involved in *N*-linked oligosaccharide degradation. GH19 PPTG_17667 expression was up-regulated 11-fold between 36 and 60 hpi and was the 12th most highly expressed *P*. *parasitica* CWDE gene (S6 Table). The sole GH38 gene had the 14th highest expression level. The GH109 and GH123 genes encoding enzymes that can attack *O*-linked oligosaccharides were predominantly expressed during middle and late infection (S7 Fig). GH109 enzymes cleave α-1,3-linkages between *N*-acetylgalactosamine residues while GH123 enzymes cleave the β-1,3-linkage between *N*-acetylgalactosamine and the terminal galactose residue of the oligosaccharide chain. Three GH109 genes were in the top 20% of CWDEs in terms of overall transcript abundance.

## Discussion

In the majority of cases, the establishment of plant disease by fungal and Oomycete pathogens requires penetration of the plant cell wall. Plant cell walls are highly complex structures that play a number of important roles during plant growth and development. In young, growing tissues, they must be strong enough to allow generation of the turgor pressure that drives cell expansion but at the same time they must be flexible enough to yield to this pressure and have properties that will regulate the direction of cell expansion. After growth has stopped, intrusion of additional components, notably lignin, inhibits wall extension and increases wall strength.

In addition to these fundamental roles in normal growth and development, plant cell walls also function as major barriers against invasion by potential pathogens. To break through this barrier, pathogens secrete a wide variety of enzymes that together are able to digest the diversity of complex cell wall polysaccharides. Early biochemical research on pathogen CWDEs documented the secretion of pectinases, hemicellulases and cellulases during infection of plant tissues and explored the substrate specificities of selected enzymes [56–59]. More recently, CWDEs and other carbohydrate active proteins have been classified into CAZyme families based on their amino acid sequence. This has been a major advance for studies of the metabolism and catabolism of complex carbohydrates. However, a single CAZyme family may contain proteins with a range of catalytic and substrate specificities and detailed sequence comparisons with proteins of known function are required in order to determine likely specificities of individual proteins. This form of intensive analysis has been applied in order to characterise the complement of CWDE genes in the *P*. *parasitica* genome [26].

The advent of modern molecular techniques has allowed elucidation of the size of CWDE CAZyme families in plant pathogens and investigations of the expression of their genes. Until relatively recently, most gene expression studies used ESTs, microarrays or PCR-based approaches but the development of next—generation sequencing, in particular the RNA-Seq approach, has dramatically increased our ability to determine gene expression patterns under selected conditions. RNA-Seq has now been applied to a number of plant-pathogen systems to gain an understanding of changes in the expression of pathogen and/or plant genes during infection. In the current study, we applied the RNA-Seq approach to determine patterns of expression of *P*. *parasitica* CWDE genes during the establishment of disease in lupin seedlings. The results show that 12,962 of the 20,403 *P*. *parasitica* predicted transcripts are expressed during the first 60 h after inoculation and that the transcriptomes include 278 of the 431 known *P*. *parasitica* CWDEs [26].

**Fig 4:**
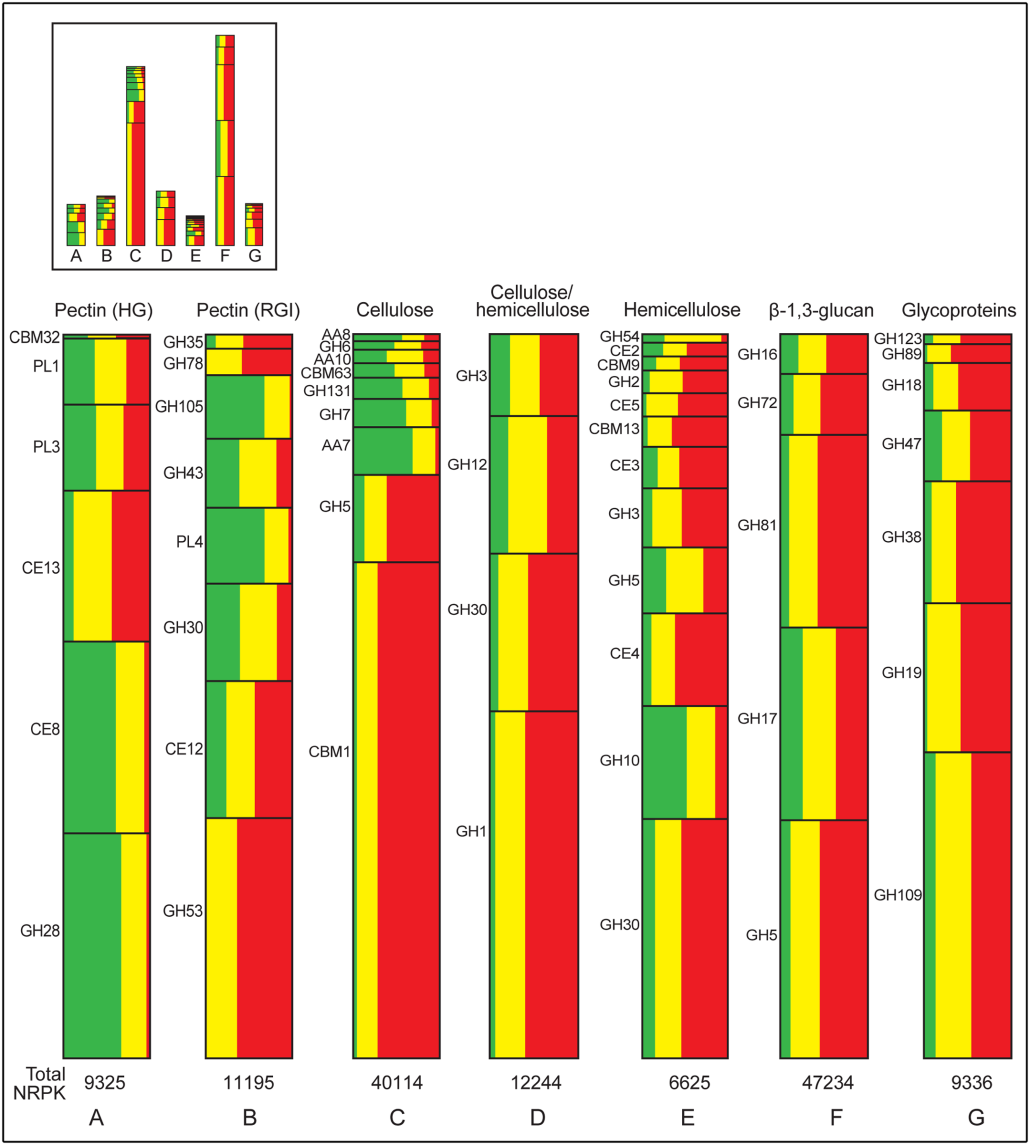
Representation of the percentage of NRPK counts for each CWDE family during early (green, 30–36 hpi), middle (yellow, 42–48 hpi) and late (red, 54–60 hpi) infection of lupin roots as a percentage of the total number of NRPK counts from all CWDEs predicted to target a particular substrate. The total number of NRPK counts for each substrate are shown below the columns. The inset at the top shows the relative heights (transcript abundance) for the different substrate categories. HG: homogalacturonan; RGI: rhamnogalacturonan I.

Our RNA-Seq analysis of *P*. *parasitica* CWDE transcriptomes in infected lupin roots has documented the predominant expression of pectinase genes during early infection, the strong expression of genes targeting cellulose and/or hemicelluloses, β-1,3-glucans and glycoproteins during middle and late infection and the dominance of cellulose-directed CBM1 transcripts during late infection (Fig 4). The results provide abundant evidence of a cascade of CWDE gene expression associated with the degradation of these five major categories of plant cell wall components.

In combination with prior gene-by-gene analysis of the potential function of each CWDE in the *P*. *parasitica* genome [26], it has also been possible to extract information from the RNA-Seq data that gives indications of possible expression cascades of genes whose encoded proteins target the same or closely related polysaccharides. If the gene expression profiles are interpreted as being indicative of the levels of enzyme production and activity, several general trends can be derived from the data. (i) Backbone chains of complex carbohydrates are targeted before side chains; (ii) internal linkages within the backbone are often targeted before terminal residues; (iii) demethylation precedes deactylation; (iv) digestion of cellulosic β-1,4-glucan chains is preceded by disruption of cellulose microfibrils by proteins with auxiliary activities and non-catalytic, binding properties; (v) high levels of β-1,3-glucanases may digest plant-derived callose produced during basal defence; and (vi) wall glycoproteins are degraded late in infection.

### Degradation of pectins

#### A cascade of gene expression during pectin degradation

Genes from GH28, CE8, GH105 and PL4 CAZyme families of pectinases are predominantly expressed during early stages of lupin root infection (Figs 3 and 4). GH28 and CE8 proteins target the backbone of homogalacturonan, typically the most abundant pectin in plant cell walls. GH105 and PL4 proteins target the backbone of RGI. During the middle and late stages of infection, predominant pectinase gene expression changes to those encoding enzymes that remove acetyl groups from galacturonic acid residues, that cleave terminal residues in the pectin backbone or that attack RG1 side chains.

A little over 30% of the total NRPK counts from genes encoding enzymes that act on homogalacturonan are endopolygalacturonases belonging to the GH28 family. Although it has been notoriously difficult to demonstrate an essential role for pathogen CWDEs in disease development, a requirement for polygalacturonases for tissue maceration and pathogen virulence has now been documented in a number of pathosystems [10]. Production of polygalacturonases early in infection is often observed [57,59–61]. During early stages of infection of the lupin roots by *P*. *parasitica*, GH28 polygalacturonases and CE8 pectin methyl esterases constitute 31% and 27% of the homogalacturonan-directed transcript pool, respectively. These two families have similar transcript profiles over the 60-h infection time-course, a feature consistent with their synergistic action as seen in a number of plant-fungal pathogen interactions [39,62–64]. Often, polygalacturonases preferentially degrade unesterified homogalacturonan and removal of the methyl group by pectin methyl esterases is thought to improve access of the polygalacturonase to the α-1,4-linkage between the galacturonic acid residues [62,65–67].

As seen for homogalacturonan degradation, genes encoding proteins that target the RGI backbone, namely GH105 rhamnogalacturonyl hydrolase and PL4 rhamnogalacturonan lyases, were predominantly expressed early in infection (Figs 3 and 4, S2 Fig). Similar expression profiles of GH28, PL4 and GH105 genes also occur during infection of rice by *Rhizoctonia solani* [68]. *P*. *parasitica* GH105 transcript abundance is only a third of that of the homogalacturonan-directed GH28 family, a likely reflection of the fact that homogalacturonan is typically the major pectin component in plant cell walls [69]. High levels of expression of GH105 genes during *Colletotrichum higginsianum* infection of Arabidopsis [39] and of PL4 genes during *R*. *solani* infection of rice [68] suggest that RGI degradation is an important part of disease development in these pathosystems.

In contrast to the early production of pectin methyl esterases, *P*. *parasitica* pectin acetyl esterases are predominantly expressed during middle and late phases of lupin root infection (Fig 4). CE13 acetyl esterases act on homogalacturonan while CE12 acetyl esterases act on both homogalacturonan and RGI. Deacetylation is thought to be required for the degradation of pectins by other enzymes [70,71] and consequently it might be expected that pectin acetyl esterases should be produced early in infection. However, the presence of methyl groups can hinder the action of acetyl esterases [72]. Typically pectins are highly methylated but sparsely acetylated [72] thus the removal of methyl groups from galacturonic acid residues may need to occur before deacetylation by the CE12 and CE13 acetyl esterases allows the complete hydrolysis of lupin cell wall pectins.

During lupin root infection, two families of lyases that act on the non-reducing end terminal galacturonic acid residues of homogalacturonan, PL1 and PL3, are, overall as a family, constitutively expressed (Fig 4). PL1 pectin lyases prefer methyl esterified residues [62] but the pectate lyases from PL1 and PL3 can act on homogalacturonans with low amounts of esterification [73]. Removal of methyl esters from homogalacturonan by CE8 enzymes early in infection may make it more susceptible to attack by PL3 lyases and CE12 and CE13 acetyl esterases. During *Zymoseptoria tritici* infection of wheat, transcript levels for both PL1 and PL3 genes also peak after those of CE8 [74].

The expression of GH78 genes which encode enzymes that cleave rhamnose residues from the non-reducing end of the RGI backbone occurs only during middle and late infection. The timing of GH78 gene expression is appropriate given that cleavage of the RGI backbone by GH105 hydrolases early in infection will create more fragment ends upon which the GH78 enzymes can act. Both galactose and rhamnose can induce GH78 expression [75,76].

Proteins from *P*. *parasitica* GH30, GH35, GH43 and GH53 families are involved in the degradation of the side chains of RGI. These GH30 β-1,6-galactanases and GH43 α-1,5-arabinases genes are predominantly expressed during early and middle phases in the infection time-course while the GH35 β-1,6-galactanase and the GH53 β-1,4-galactanases are predominantly (GH35) or exclusively (GH53) expressed during middle and late infection. Transcripts from three GH53 β-1,4-galactanase genes constitute over 30% of the RGI-directed pectinases transcript pool (Fig 4).

### Degradation of cellulose and hemicellulose

Degradation of cellulose occurs through cleavage of internal linkages within the β-1,4-glucan backbone by endo-β-1,4-glucanases, removal of terminal residues by exo-β-1,4-glucanases and degradation of cellobiose by β-glucosidases (reviewed in [77]). Cellulose degradation may also require digestion of hemicellulose molecules that are closely associated with the cellulose microfibrils [78]. Some CAZymes act on cellulose and hemicellulose but often different enzymes are required to degrade the hemicellulose backbone (e.g. GH10 β-1,4-xylanases and GH30 β-1,4-xylosidases and β-1,4-xylanases) and side chains [48,79]. During lupin root infection, cellulase and hemicellulase genes are predominantly expressed late in infection, after the early expression of a major cohort of pectinase genes (Fig 4). This fits with the facts that pectins can mask cellulose and hemicellulose epitopes [80,81] and that pectin degradation usually precedes that of hemicellulose and cellulose [57,59,82]. The expression profiles of *P*. *parasitica* cellulase and hemicellulase genes also provide clues as to the order in which particular CAZymes may act on cellulose and hemicellulose substrates.

#### A cascade of gene expression during cellulose and hemicellulose degradation

Although the highest levels of cellulase and hemicellulase transcripts are seen during middle and late infection, some families whose encoded proteins target cellulose and/or hemicellulose are predominantly expressed early in infection, albeit at only about 20% of the level of those peaking at middle-late infection (Figs 2–4). The genes whose expression peaks early encode gluco-oligosaccharide oxidases (AA7), iron reductases (AA8), copper-dependent lytic polysaccharide mono-oxygenases (AA10), endo-β-1,4-glucanases (GH6, GH7, GH131) and β-1,4-xylanases (GH10). AA7, AA8 and AA10 enzymes can disrupt bonds within cellulose microfibrils and cleave cellulose chains, either independently by changing the redox environment or in conjunction with endoglucanases [42,83,84]. Their early production and action could make cellulose and hemicellulose more susceptible to digestion by other enzymes. The early expression of GH6 and GH7 endoglucanase and GH10 endoxylanase genes whose encoded proteins cleave cellulose and hemicellulose backbones is also appropriate because the activity of these enzymes will amplify the number of backbone fragments upon which exoglycanases can act. Early expression of GH6 and GH7 genes also occurs during *P*. *parasitica* infection of other hosts and in other plant-pathogen interactions [31,68,85]. GH6 and GH7 CAZymes may act in concert with AA proteins to begin digestion of cellulose glucan chains. The early expression of GH10 endo-β-1,4-xylanases is consistent with studies showing that breakdown of hemicellulose by GH10 xylanases facilitates degradation of cellulose [78,86]. GH10 genes in *Magnaporthe oryzae* are expressed 24 hpi of wheat leaves and are essential for pathogenicity [17].

The cellulose- and hemicellulose-directed hydrolase genes with the highest levels of transcript abundance are from GH1, GH3, GH5, GH7, GH12 and GH30 CAZyme families (Fig 4). GH5 and GH12 proteins are endoglucanases, GH1 and GH3 proteins are exoglucanases or enzymes targeting disaccharides, and GH30 proteins are exoglucanases and endoxylanases. They are predominantly expressed during middle and late infection. The timing of expression of these genes is consistent with the production of their encoded enzymes after initial degradation of the cellulose microfibrils and dismantling of the pectin network. GH1 genes are also expressed late in the infection of Arabidopsis by *P*. *parasitica* [31].

It has been suggested that enzymatic digestion of hemicellulose backbones may be hindered by the presence of side chains and by the addition of acetyl, methyl or ferulic acid groups on backbone residues [87–90]. In support of this hypothesis, *Aspergillus* GH10 and GH11 enzymes have been shown to degrade hemicelluloses more effectively in the presence of an α-L-arabinofuranosidase (GH54) that attacks side chains on targeted hemicellulose molecules [90]. Similarly, a GH10 from *Thermoascus aurantiacus* works more efficiently in the presence of a feruloyl esterase [91]. Chemical deacetylation of wall material also enhances hemicellulose degradation [92]. During the lupin-*P*. *parasitica* interaction, the timing of expression of *P*. *parasitica* genes whose encoded proteins target hemicellulose side chains or ester linkages is also consistent with their proposed role in enhancing the exposure of the hemicellulose backbone to degradation. α-L-arabinofuranosidases from GH43 and GH54 families are mainly expressed during early and middle infection as are genes encoding acetyl xylan esterases (CE2, CE3, CE4, CE5) (Fig 4).

#### Dominance of cellulose-directed CBM1 late in infection

Cellulose microfibrils in plant cell walls consist of 30–36 tightly packed, highly cross-linked chains of β-1,4-glucans surrounded by hemicelluloses and, in secondary walls, embedded in lignin [93]. Cellulose chains in intact, insoluble microfibrils are highly inaccessible to cellulase enzymes. One of the processes by which cellulose chains, especially those not on the microfibril surface, are exposed for cellulase attack is through the action of CBMs. In fungi, cellulose-specific CBM1 domains are often present together with endoglucanase domains in CAZyme proteins [44,64,94,95]. It is believed that the CBMs act to concentrate enzyme activity at its substrate, influence the specificity of the catalytic domain and/or disrupt the structure of the substrate, opening it up to attack by the catalytic domain within the same protein [93,96–99].

The *P*. *parasitica* genome contains two CBM families, CBM1 and CBM63, whose encoded proteins target cellulose [26,45]. They have 17 and 12 members, respectively [26]. In contrast to the situation in many fungi and bacteria, in *P*. *parasitica* only about 25% of the CBM-containing proteins also contain a catalytic CAZyme domain detected by the automated CAZyme prediction programmes dbCAN (http://csbl.bmb.uga.edu/dbCAN/) and CAT (http://mothra.ornl.gov/cgi-bin/cat/cat.cgi), a situation seen in other *Phytophthora* species [26,47,100]. There are no CAZyme catalytic domains in the *P*. *parasitica* CBM1 or CBM63 proteins. Bioinformatic analysis using domain recognition algorithms on NCBI shows that only one of the expressed CBM1 or CBM63 genes contain a catalytic domain of any type (LM Blackman, unpublished observations); CBM1 PPTG_19721, a gene expressed at a low level, contains an acetyltransferase domain.

CBM63 proteins have expansin-like properties that produce a relaxation of cellulose microfibrils, and their production by fungal and bacterial phytopathogens suggests they play a role in plant cell wall degradation [101]. Although 10 of the 12 *P*. *parasitica* CBM63 genes are expressed during lupin root infection, CBM63 transcript levels are low and relatively constant (Fig 4). Six *P*. *parasitica* CBM63 proteins contain a C-terminal TMD [26]. Together these data suggest that the *P*. *parasitica* CBM63 enzymes may be involved more in adjusting the extensibility of the *Phytophthora* cell walls than in loosening the cellulosic framework of the lupin cell walls.

In contrast to the potentially minor role played by CBM63 proteins during lupin root infection, based on their transcript abundance, *P*. *parasitica* CBM1 proteins play a major role in plant colonisation and disease development. A CBM1 gene, PPTG_06045, that contains two CBM1 modules, is the most highly expressed *P*. *parasitica* CWDE gene (Figs 3 and 4 and S6 Table) and 20% of the total NRPK counts for all CWDE genes come from the CBM1 family. CBM1 PPTG_06045 and two other highly transcribed CBM1 genes are expressed predominantly during late infection (Figs 3 and 4 and S6 Table). *P*. *parasitica* CBM1 genes and *Aspergillus niger* CBM1-containing CAZyme genes are also expressed late in the infection of Arabidopsis [31] and the degradation of wheat straw [95], respectively. The *P*. *parasitica* CBM1 protein that has the second highest expression level, PPTG_13482, is an elicitor of the plant defence response in tobacco [102].

Given that the CBM1 modules do not co-exist with catalytic domains in *P*. *parasitica* CAZymes, evidence of their potential function was sought by comparing the expression profiles of the CBM1 family with those of families encoding catalytic cellulases. In fungal phytopathogens, CBM1 domains frequently occur in proteins containing catalytic GH5, GH6, GH7 and GH131 domains and, less frequently, with AA8, AA9 and AA10 subunits [42,77,94,103]. Our RNA-Seq data show that the high levels of CBM1 transcripts late in infection do not coincide with the peaks in expression of GH6, GH7, GH131, AA8 or AA10 genes early during infection, suggesting that it is unlikely that CBM1 plays a role in early cellulose degradation by GH6, GH7 or GH131 β-1,4-glucanases, AA8 reductases or AA10 monooxygenases.

The cellulose-directed *P*. *parasitica* glucanases that do have similar expression profiles to that of CMB1 belong to the GH1, GH5 and GH30 families. To our knowledge, there is no published evidence of synergistic action of CBM1 modules with the exo-β-1,4-glucanase activity of GH1 and GH30 enzymes. This leaves the GH5 endoglucanase CAZyme family as potential partners for CBM1 action in the lupin-*P*. *parasitica* interaction. Although the total number of cellulose-directed GH5 transcripts is only about 30% of that for CBM1, their expression profiles are similar, both peaking late in infection. Co-expression of *R*. *solani* GH5 and CBM1 genes has been observed during the infection of rice [68] and in fungi there are many examples of GH5 and CBM1 modules existing in the same protein [44]. Perhaps an abundance of CBM1 proteins generate widespread exposure of cellulose chains upon which GH5 endoglucanases can act and effectively degrade cellulose in the lupin cell walls.

### Degradation of β-1,3-glucans

A striking feature of the *P*. *parasitica* CWDE transcriptome during lupin root infection is the magnitude of the response directed towards the degradation of β-1,3-glucans during middle and late phases of infection (Figs 2–4). The cohort is dominated by genes encoding putative exo-acting β-1,3-glucosidases from the GH5 and GH17 families and endo-β-1,3-glucanases from the GH81 family. The two most highly expressed β-1,3-glucanases were a GH5 gene containing a putative β-1,3-glucan binding module CBM43 and a GH81 gene. During infection of tanoak by *P*. *ramorum*, β-1,3-glucanase genes from GH16, GH17 and GH81 families accounted for 35% of all CWDE transcripts [104].

Pathogen β-1,3-glucanases target callosic β-1,3-glucans that are rapidly deposited by the plant in cell wall appositions, or papillae, at infection sites. Papilla formation is a central part of the basal plant defence response to invading fungal, oomycete and bacterial pathogens during non-host, incompatible and compatible interactions [105] and callose is a major component of these specialised wall structures [105,106]. Papillae do not always successfully inhibit pathogen ingress but it is believed they provide a physical barrier that at least slows down pathogen penetration, thereby giving the plant more time to mobilise other aspects of their defence response [107]. Callose is thought to provide a structural framework for other wall polymers, to protect other wall components from degradation and to inhibit pathogen toxin or effector entry [108]. The speed of callose deposition appears to be important. It is affected by environmental conditions and the identity of pathogen elicitors [109]. It may be faster during an incompatible interaction than during a compatible interaction [110,111]. It is also possible that the apparently delayed appearance of callose during compatible interactions may be the result of a virulent pathogen’s ability to degrade callose as it is formed. The infrequent observation of callose around *P*. *parasitica* haustoria in the infected lupin roots (P Torreña, K Kots, LM Blackman and AR Hardham, unpublished observations) may be due to *P*. *parasitica* β-1,3-glucanase activity. In contrast to the situation in *P*. *parasitica* and *P*. *ramorum*, during infection of cucumber by *Pseudoperonospora cubensis*, β-1,3-glucanase genes from GH16 and GH17 families were not expressed [85]. *P*. *ramorum* and *P*. *parasitica* are hemibiotrophic pathogens while *Ps*. *cubensis* is a biotroph. It is possible that the abundance of β-1,3-glucanase gene transcripts in the *P*. *ramorum* and *P*. *parasitica* transcriptomes may be influenced by the nature of the interaction between plant and pathogen, with lower levels of callose deposition occurring during the biotrophic interaction than during the hemibiotrophic interactions, leading to lower levels of β-1,3-glucanase gene transcription.

The fact that *Phytophthora* and other Oomycete species have β-1,3-glucans in their cell walls and in vesicular storage compartments [110,112,113] must also be taken into account when considering potential functions of *P*. *parasitica* β-1,3-glucanases. The *P*. *parasitica* β-1,3-glucanases that have TMDs or GPI anchors could, for example, be located in the pathogen plasma membrane and involved in modifying the *P*. *parasitica* hyphal wall. However, in *P*. *infestans*, different GH5 β-1,3-glucanase genes are expressed *in planta* compared to *in vitro* [114] and in the lupin-*P*. *parasitica* interaction, the high level of expression of β-1,3-glucanase genes that lack a membrane anchor during middle and late infection seems more consistent with a role in the degradation of plant callose than with pathogen wall remodelling or endogenous nutrient mobilisation. Immunolocalization of fungal β-1,3-glucanases at host-pathogen interfaces has been interpreted as being indicative of secretion of β-1,3-glucanases at sites of callose deposition [115,116].

### Degradation of glycoproteins

Glycoproteins serve many functions in eukaryotic cells. Within the plant cell wall, they contribute to wall structure by crosslinking wall polysaccharides, and their degradation affects interactions between pectins, cellulose and hemicelluloses, and can enhance nutrient release [1,117–119].

*P*. *parasitica* genes encoding proteins with the potential to degrade plant cell wall glycoproteins are most strongly expressed during the advanced stages of lupin infection, as has been observed during the infection of Arabidopsis by *P*. *parasitica* [31] and of cucumber by *P*. *cubensis* [85]. The putative glycoprotein-directed transcript pool is dominated by GH19 *N*-acetylglucosaminidases, GH38 α-mannosidases and GH109 *N*-acetylgalactosaminidases. *N*-acetylglucosamine linkages also occur in chitin [120] but, as neither plant nor *Phytophthora* cell walls contain chitin [1,112], it seems likely that the GH18, GH19 and GH89 *N*-acetylgalactosaminidases encoded by the genomes of *Phytophthora*, *Pythium* and *Saprolegnia* species ([26]; Saprolegnia Genome Sequencing Project, Broad Institute of Harvard and MIT: http://www.broadinstitute.org/,[27,121]) act on plant or pathogen glycoproteins. Although *N*-acetylgalactosaminidases have been identified in the genomes of a number of plant pathogens, there is still little information on their role during plant infection [26,27,39,122]. The GH47 α-mannosidase genes in the *P*. *parasitica* genome were expressed throughout the infection time-course, as also observed during the cucumber-*Ps*. *cubensis* interacti on [85] and it is possible that the GH38 enzymes function in glycoprotein maturation within the *P*. *parasitica* secretory pathway.

## Supporting Information

S1 Fig Normalized reads per kilobase (NRPK) after transformation of the RNA-Seq data by DESeq for five *P*. *parasitica* genes used as reference genes for qPCR data normalization [37]. WS021: 40S ribosomal protein S3A (PPTG_07764), UCE: ubiquitin-conjugating enzyme (PPTG_08273), PPI: peptidyl prolyl isomerase 2 (PPTG_02092), WS041 (PPTG_09948) and PLA2: phospholipase A2 (PPTG_08636). The fold change between the highest and lowest values across the time-course is shown in the legends. **(A)** Mean NRPK values and standard deviations of three biological replicates. **(B)** Median NRPK values of three biological replicates.
(TIF)

S2 Fig Pectinase expression profiles during lupin root infection. The diagrams show representations of the structures of homogalacturonan and RGI and putative sites of activity of pectinases from 14 CAZyme families. The vertical axis of each graph shows the total median NRPK values from the multiple location data set for all members of a particular CAZyme family whose highest level of expression occurs at early (30 or 36 hpi), middle (42 or 48 hpi) or late (54 or 60 hpi) stages of infection. The horizontal axis shows the hpi. The number of genes contributing to the data in each graph, the total number of expressed genes in that CAZyme family and the total number of genes in the CAZyme family in the *P*. *parasitica* genome are indicated below by the three values separated by slashes. Only genes that had a total NRPK value over the time-course ≥50 have been included. The graphs are arranged in order of decreasing levels of transcript abundance. Some CAZyme families have multiple putative substrate targets: ^i^proteins that may act on pectins and glycoproteins; ^ii^proteins that may act on pectins, hemicellulose and glycoproteins. Early: GH28, 7/8/18; CE8, 6/9/15; GH105, 1/1/1; PL4, 3/3/6; PL3, 3/11/17; GH30, 2/19/21; GH43, 3/6/7; PL1, 3/9/21; CE12, 2/11/14 and CE13, 1/5/6. Middle: GH30, 2/19/21; CE13, 1/5/6; GH43, 2/6/7; GH78, 1/4/4; CE12, 3/11/14 and PL1, 1/9/21. Late: GH53, 2/3/6; CE12, 5/11/14; CE13, 2/5/6; PL3, 4/11/17; PL1, 1/9/21; GH35, 1/1/1; GH78, 1/4/4; GH43, 1/6/7 and CBM32, 1/1/1.
(TIF)

S3 Fig Cellulase expression profiles during lupin root infection. The diagrams show a representation of cellulose structure and putative sites of activity of proteins from nine CAZyme families. The vertical axis of each graph shows the total median NRPK values from the multiple location data set for all members of a particular CAZyme family whose highest level of expression occurs at early (30 or 36 hpi), middle (42 or 48 hpi) or late (54 or 60 hpi) stages of infection. The horizontal axis shows the hpi. The number of genes contributing to the data in each graph, the total number of expressed genes in that CAZyme family and the total number of genes in the CAZyme family in the *P*. *parasitica* genome are indicated below by the three values separated by slashes. Only genes that had a total NRPK value over the time-course ≥50 have been included. The graphs are arranged in order of decreasing levels of transcript abundance. Some CAZyme families have multiple putative substrate targets: ^iii^proteins that may act on cellulose and β-1,3-glucans. Early: AA7, 4/4/5; GH7, 2/2/5; GH131, 2/3/5; CBM63, 2/10/12; AA10, 1/4/4; GH6, 2/6/7 and AA8, 2/3/3. Middle: AA10, 2/4/4; CBM63, 2/10/12; GH131, 1/3/5; GH5, 1/20/25 and GH6, 1/6/7. Late: CBM1, 11/14/17 and GH5, 9/20/25.
(TIF)

S4 Fig Cellulase and hemicellulase expression profiles during lupin root infection. The diagrams show representations of cellulose and the hemicelluloses xyloglucan and glucuronoxylan and putative sites of activity of proteins from four CAZyme families. The vertical axis of each graph shows the total median NRPK values from the multiple location data set for all members of a particular CAZyme family whose highest level of expression occurs at early (30 or 36 hpi), middle (42 or 48 hpi) or late (54 or 60 hpi) stages of infection. The horizontal axis shows the hpi. The number of genes contributing to the data in each graph, the total number of expressed genes in that CAZyme family and the total number of genes in the CAZyme family in the *P*. *parasitica* genome are indicated below by the three values separated by slashes. Only genes that had a total NRPK value over the time-course ≥50 have been included. The graphs are arranged in order of decreasing levels of transcript abundance. Some CAZyme families have multiple putative substrate targets: ^iv^proteins that may act cellulose, hemicellulose and glycoproteins. Early: GH12, 1/6/15 and GH3, 2/16/25. Middle: GH30, 3/19/21; GH12, 2/6/12 and GH3, 1/16/25. Late: GH1, 12/15/17; GH30, 4/19/21; GH12, 1/6/12 and GH3, 6/16/25
(TIF)

S5 Fig Hemicellulase expression profiles during lupin root infection. The diagrams show representations of the hemicelluloses xyloglucan, glucuronoxylan and galactomannan/galactoglucomannan and putative sites of activity of proteins from 12 CAZyme families. The vertical axis of each graph shows the total median NRPK values from the multiple location data set for all members of a particular CAZyme family whose highest level of expression occurs at early (30 or 36 hpi), middle (42 or 48 hpi) or late (54 or 60 hpi) stages of infection. The horizontal axis shows the hpi. The number of genes contributing to the data in each graph, the total number of expressed genes in that CAZyme family and the total number of genes in the CAZyme family in the *P*. *parasitica* genome are indicated below by the three values separated by slashes. Only genes that had a total NRPK value over the time-course ≥50 have been included. The graphs are arranged in order of decreasing levels of transcript abundance. Some CAZyme families have multiple putative substrate targets: ^ii^proteins that may act on pectins, hemicellulose and glycoproteins; ^v^proteins that may act on hemicellulose and glycoproteins. Early: GH10, 2/4/4. Middle: GH5, 2/20/25; GH10, 1/4/4; GH30^a^, 1/ 19/21 and GH54, 1/1/1. Late: GH30^b^, 4/19/21; CE4, 2/2/2; GH3, 1/16/25; CE3, 1/1/1; CBM13, 1/3/4; CE5, 2/4/4; GH2, 1/1/1; CBM9, 1/1/1; CE2, 1/1/1. ^a^This GH30 also contains a CBM13 domain. ^b^Three of these GH30 proteins also contain a CBM13 domain.
(TIF)

S6 Fig β-1,3-glucanase expression profiles during lupin root infection. The diagram shows a representation of the structure of β-1,3-glucan and putative sites of activity of proteins from five CAZyme families. The vertical axis of each graph shows the total median NRPK values from the multiple location data set for all members of a particular CAZyme family whose highest level of expression occurs at early (30 or 36 hpi), middle (42 or 48 hpi) or late (54 or 60 hpi) stages of infection. The horizontal axis shows the hpi. The number of genes contributing to the data in each graph, the total number of expressed genes in that CAZyme family and the total number of genes in the CAZyme family in the *P*. *parasitica* genome are indicated below by the three values separated by slashes. Only genes that had a total NRPK value over the time-course ≥50 have been included. The graphs are arranged in order of decreasing levels of transcript abundance. Some CAZyme families have multiple putative substrate targets: ^vi^proteins that may act on hemicellulose and β-1,3-glucans. Early: GH17^a^, 5/16/20 and GH72, 2/8/14. Middle: GH17, 2/16/20; GH16, 2/11/16 and GH81, 1/11/16. Late: GH5^b^, 3/20/25; GH81, 6/11/16; GH72, 6/8/14; GH17, 7/16/20 and GH16, 5/11/16. ^a^Two GH17 genes also contain a CBM13 domain. ^b^Two GH5 proteins also contain a CBM43 domain.
(TIF)

S7 Fig Expression profiles of genes targeting glycoproteins during lupin root infection. The diagrams show representations of the structures of *N*- and *O*-linked glycoproteins and putative sites of activity of enzymes from seven CAZyme families. The vertical axis of each graph shows the total median NRPK values from the multiple location data set for all members of a particular CAZyme family whose highest level of expression occurs at early (30 or 36 hpi), middle (42 or 48 hpi) or late (54 or 60 hpi) stages of infection. The horizontal axis shows the hpi. The number of genes contributing to the data in each graph, the total number of expressed genes in that CAZyme family and the total number of genes in the CAZyme family in the *P*. *parasitica* genome are indicated below by the three values separated by slashes. Only genes that had a total NRPK value over the time-course ≥50 have been included. The graphs are arranged in order of decreasing levels of transcript abundance. Middle: GH109, 2/7/7. Late: GH19, 2/2/2; GH38, 1/1/1; GH109, 5/7/7; GH47, 5/5/5; GH18, 2/2/3; GH89, 2/2/2 and GH123, 1/1/1.
(TIF)

S1 Table *P*. *parasitica* and lupin primers used for qPCR. (DOCX)

S2 Table RNA-Seq data statistics showing the total number of reads mapped per sample, the lowest Fast QC quality score across all reads in each data set, the number of reads mapped to unique locations in the *P*. *parasitica* genome and the number of reads mapped to multiple locations in the *P*. *parasitica* genome. (DOCX)

S3 Table RNA-Seq data showing raw counts for three biological replicates mapping to predicted CWDE transcripts and the normalising genes WS021: 40S ribosomal protein S3A, UCE: ubiquitin-conjugating enzyme, PPI: peptidyl prolyl isomerase 2, WS041 and PLA2: phospholipase A2. Also shown are the raw counts for the top expressed 200 genes according to the sum of the median NRPK values from the unique location data set and the top 200 differentially expressed genes. * indicates the transcripts that have been annotated as two genes in the *P*. *parasitica* genome (v2) but which are likely to be a single gene [26]. See S3 and S4 Tables for annotation of non-CWDE transcripts.
(XLSX)

S4 Table Top 200 expressed *P*. *parasitica* genes based on the sum of the median NRPK values from the uniquely mapped reads across the infection time-course. (XLSX)

S5 Table Top 200 differentially expressed *P*. *parasitica* genes ranked according to the difference between the highest and lowest median, non-zero log2 values from the unique location data set from 30 to 60 hpi. (XLSX)

S6 Table Summary of the RNA-Seq data. Shown are the putative CAZyme functions, the CAZyme family, the number of expressed genes of the total number of genes in that CAZyme family, the *P*. *parasitica* accession number, the gene rank according to the sum of median NRPK values (n = 3) from the unique location data set across the time-course, the differential expression (DE) as indicated by the ratio of the highest and lowest median non-zero log2 value from the unique location data set, the RNA-Seq NRPK values at each time point from the multiple location data set (30 hpi to 60 hpi), the sum of the median NRPK values from the multiple location data set, the sum of the median NRPK values from the unique location data set and the qPCR data for the 24 hpi to 60 hpi time points. na: qPCR data not available.
(XLSX)
