# Supplementary figures and images for: RNA-Seq Analysis of the Expression of Genes Encoding Cell Wall Degrading Enzymes during Infection of Lupin (Lupinus angustifolius) by Phytophthora parasitica
Source: PLoS One. 2015 Sep 2;10(9):e0136899. doi: 10.1371/journal.pone.0136899 (PMC4558045; doi:10.1371/journal.pone.0136899)

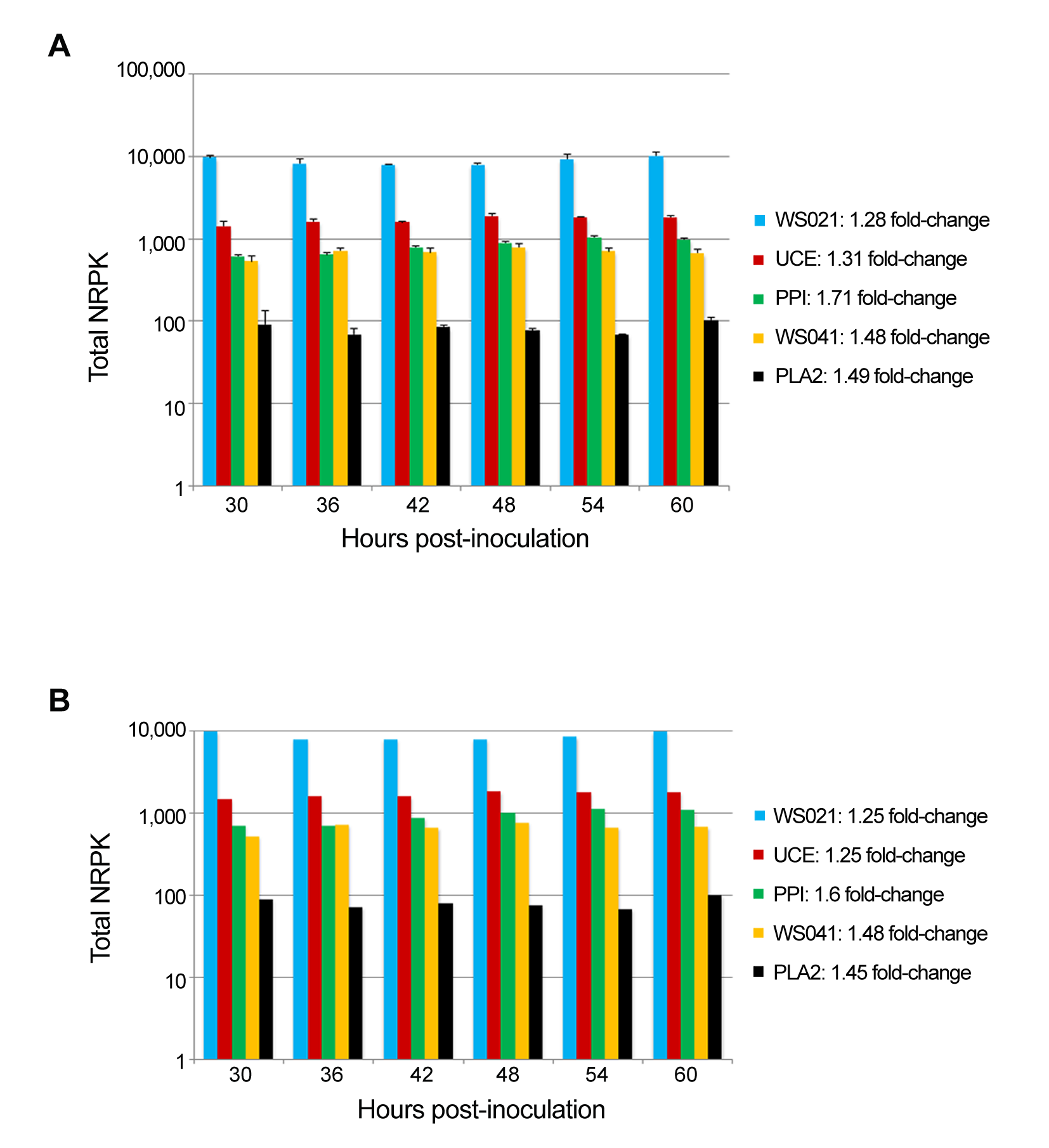

Supplement: S1 Fig — WS021: 40S ribosomal protein S3A (PPTG_07764), UCE: ubiquitin-conjugating enzyme (PPTG_08273), PPI: peptidyl prolyl isomerase 2 (PPTG_02092), WS041 (PPTG_09948) and PLA2: phospholipase A2 (PPTG_08636). The fold change between the highest and lowest values across the time-course is shown in the legends. (A) Mean NRPK values and standard deviations of three biological replicates. (B) Median NRPK values of three biological replicates. (TIF) [file pone.0136899.s001.tif]

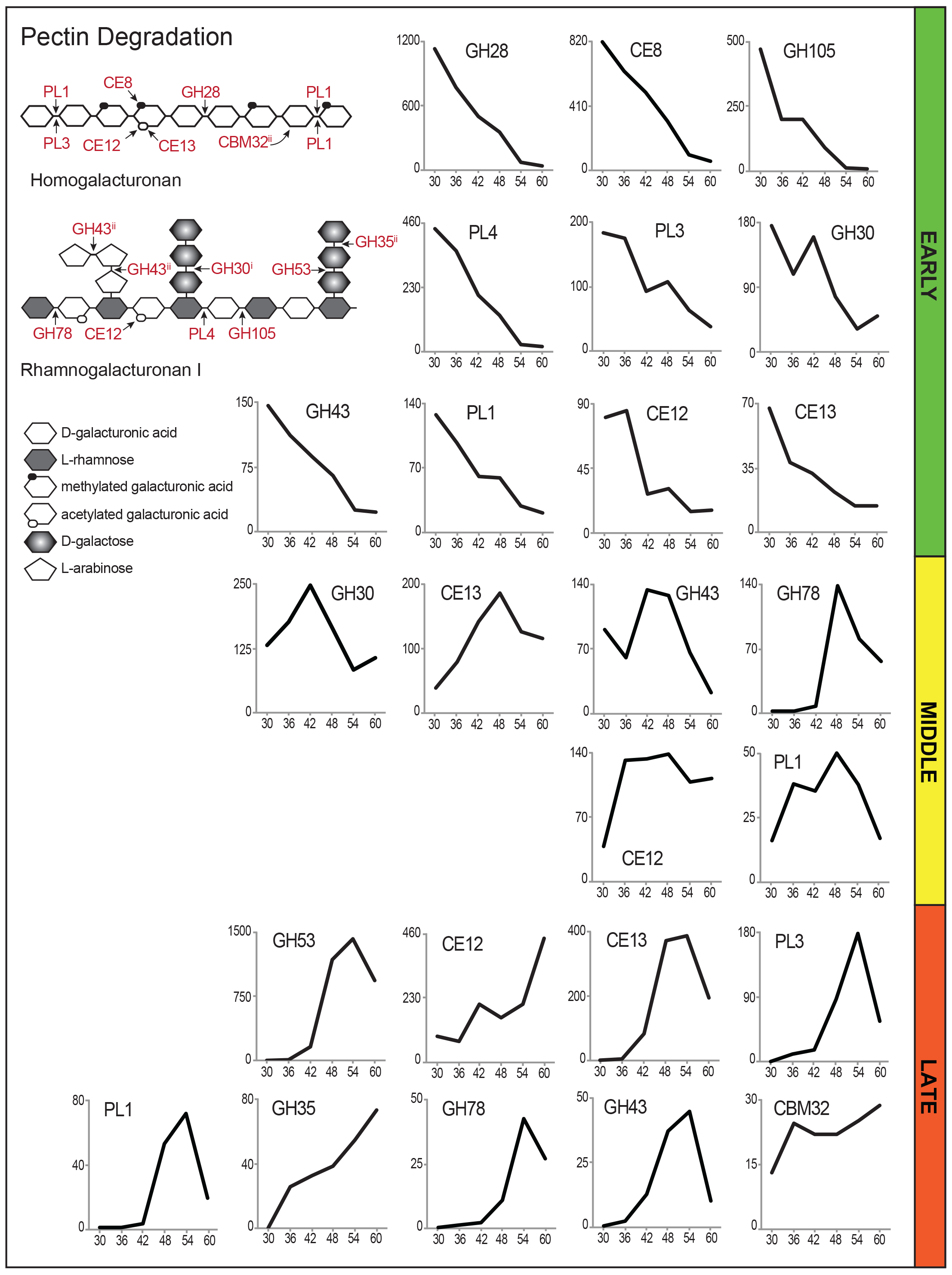

Supplement: S2 Fig — The diagrams show representations of the structures of homogalacturonan and RGI and putative sites of activity of pectinases from 14 CAZyme families. The vertical axis of each graph shows the total median NRPK values from the multiple location data set for all members of a particular CAZyme family whose highest level of expression occurs at early (30 or 36 hpi), middle (42 or 48 hpi) or late (54 or 60 hpi) stages of infection. The horizontal axis shows the hpi. The number of genes contributing to the data in each graph, the total number of expressed genes in that CAZyme family and the total number of genes in the CAZyme family in the P. parasitica genome are indicated below by the three values separated by slashes. Only genes that had a total NRPK value over the time-course ≥50 have been included. The graphs are arranged in order of decreasing levels of transcript abundance. Some CAZyme families have multiple putative substrate targets: iproteins that may act on pectins and glycoproteins; iiproteins that may act on pectins, hemicellulose and glycoproteins. Early: GH28, 7/8/18; CE8, 6/9/15; GH105, 1/1/1; PL4, 3/3/6; PL3, 3/11/17; GH30, 2/19/21; GH43, 3/6/7; PL1, 3/9/21; CE12, 2/11/14 and CE13, 1/5/6. Middle: GH30, 2/19/21; CE13, 1/5/6; GH43, 2/6/7; GH78, 1/4/4; CE12, 3/11/14 and PL1, 1/9/21. Late: GH53, 2/3/6; CE12, 5/11/14; CE13, 2/5/6; PL3, 4/11/17; PL1, 1/9/21; GH35, 1/1/1; GH78, 1/4/4; GH43, 1/6/7 and CBM32, 1/1/1. (TIF) [file pone.0136899.s002.tif]

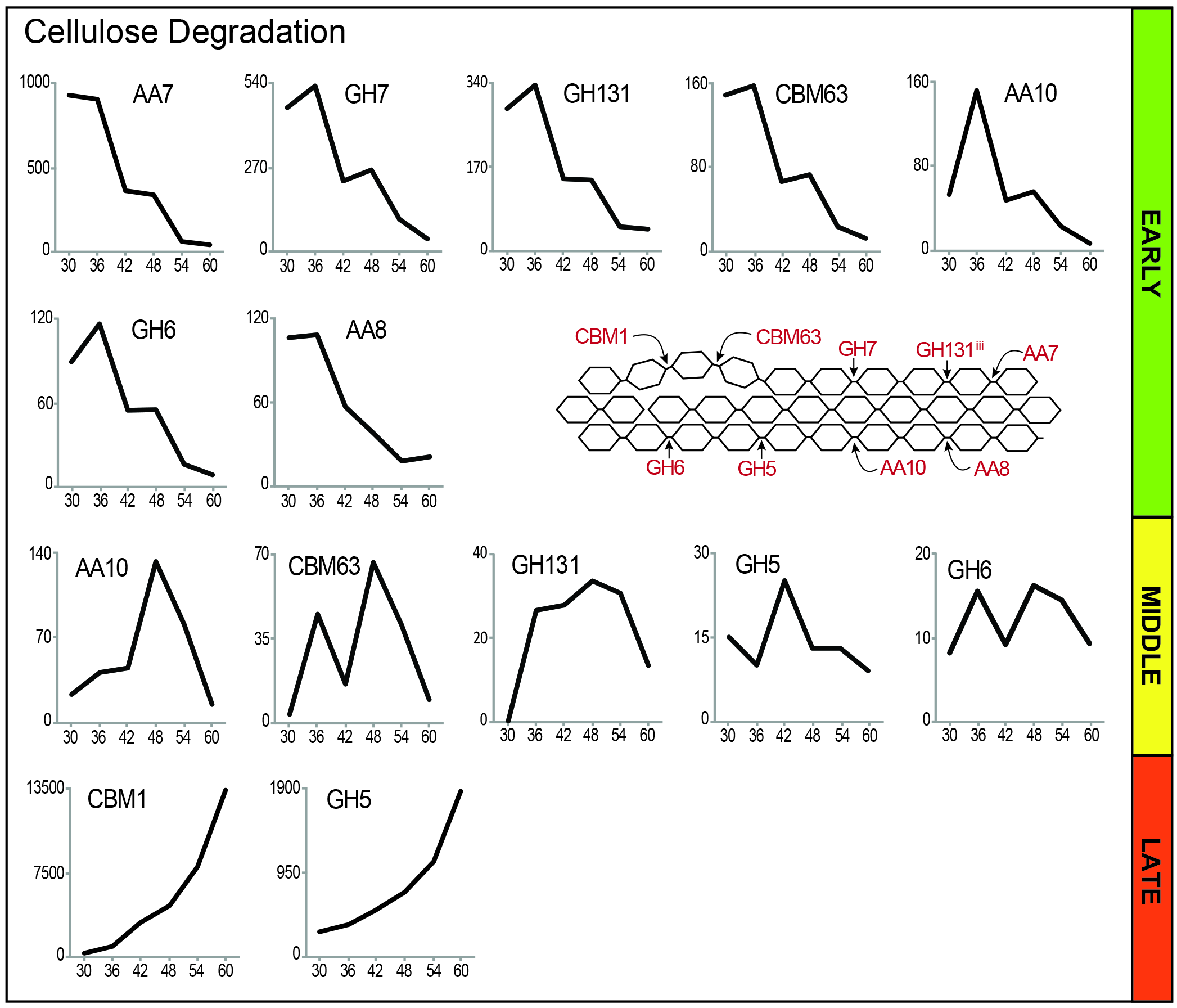

Supplement: S3 Fig — The diagrams show a representation of cellulose structure and putative sites of activity of proteins from nine CAZyme families. The vertical axis of each graph shows the total median NRPK values from the multiple location data set for all members of a particular CAZyme family whose highest level of expression occurs at early (30 or 36 hpi), middle (42 or 48 hpi) or late (54 or 60 hpi) stages of infection. The horizontal axis shows the hpi. The number of genes contributing to the data in each graph, the total number of expressed genes in that CAZyme family and the total number of genes in the CAZyme family in the P. parasitica genome are indicated below by the three values separated by slashes. Only genes that had a total NRPK value over the time-course ≥50 have been included. The graphs are arranged in order of decreasing levels of transcript abundance. Some CAZyme families have multiple putative substrate targets: iiiproteins that may act on cellulose and β-1,3-glucans. Early: AA7, 4/4/5; GH7, 2/2/5; GH131, 2/3/5; CBM63, 2/10/12; AA10, 1/4/4; GH6, 2/6/7 and AA8, 2/3/3. Middle: AA10, 2/4/4; CBM63, 2/10/12; GH131, 1/3/5; GH5, 1/20/25 and GH6, 1/6/7. Late: CBM1, 11/14/17 and GH5, 9/20/25. (TIF) [file pone.0136899.s003.tif]

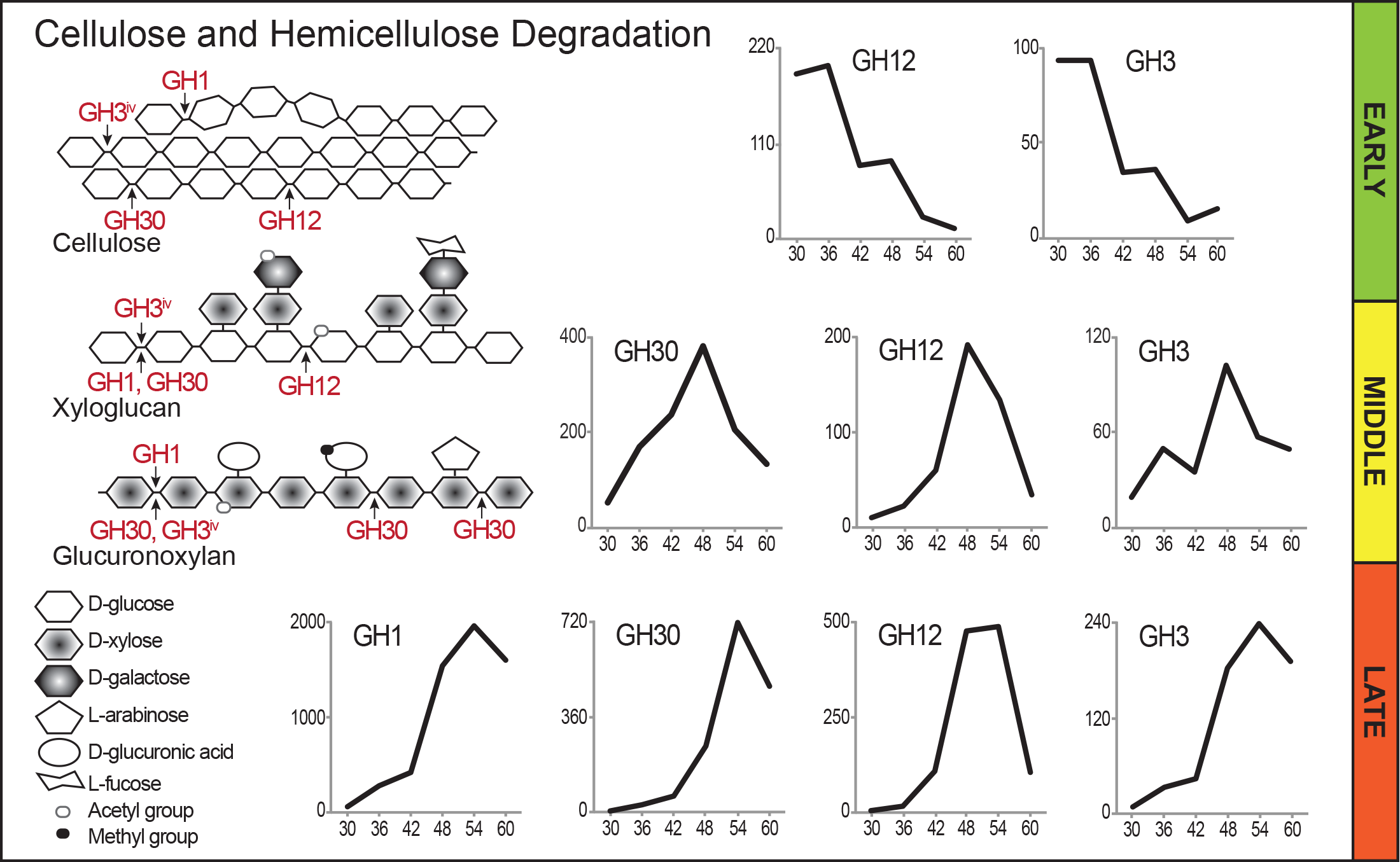

Supplement: S4 Fig — The diagrams show representations of cellulose and the hemicelluloses xyloglucan and glucuronoxylan and putative sites of activity of proteins from four CAZyme families. The vertical axis of each graph shows the total median NRPK values from the multiple location data set for all members of a particular CAZyme family whose highest level of expression occurs at early (30 or 36 hpi), middle (42 or 48 hpi) or late (54 or 60 hpi) stages of infection. The horizontal axis shows the hpi. The number of genes contributing to the data in each graph, the total number of expressed genes in that CAZyme family and the total number of genes in the CAZyme family in the P. parasitica genome are indicated below by the three values separated by slashes. Only genes that had a total NRPK value over the time-course ≥50 have been included. The graphs are arranged in order of decreasing levels of transcript abundance. Some CAZyme families have multiple putative substrate targets: ivproteins that may act cellulose, hemicellulose and glycoproteins. Early: GH12, 1/6/15 and GH3, 2/16/25. Middle: GH30, 3/19/21; GH12, 2/6/12 and GH3, 1/16/25. Late: GH1, 12/15/17; GH30, 4/19/21; GH12, 1/6/12 and GH3, 6/16/25 (TIF) [file pone.0136899.s004.tif]

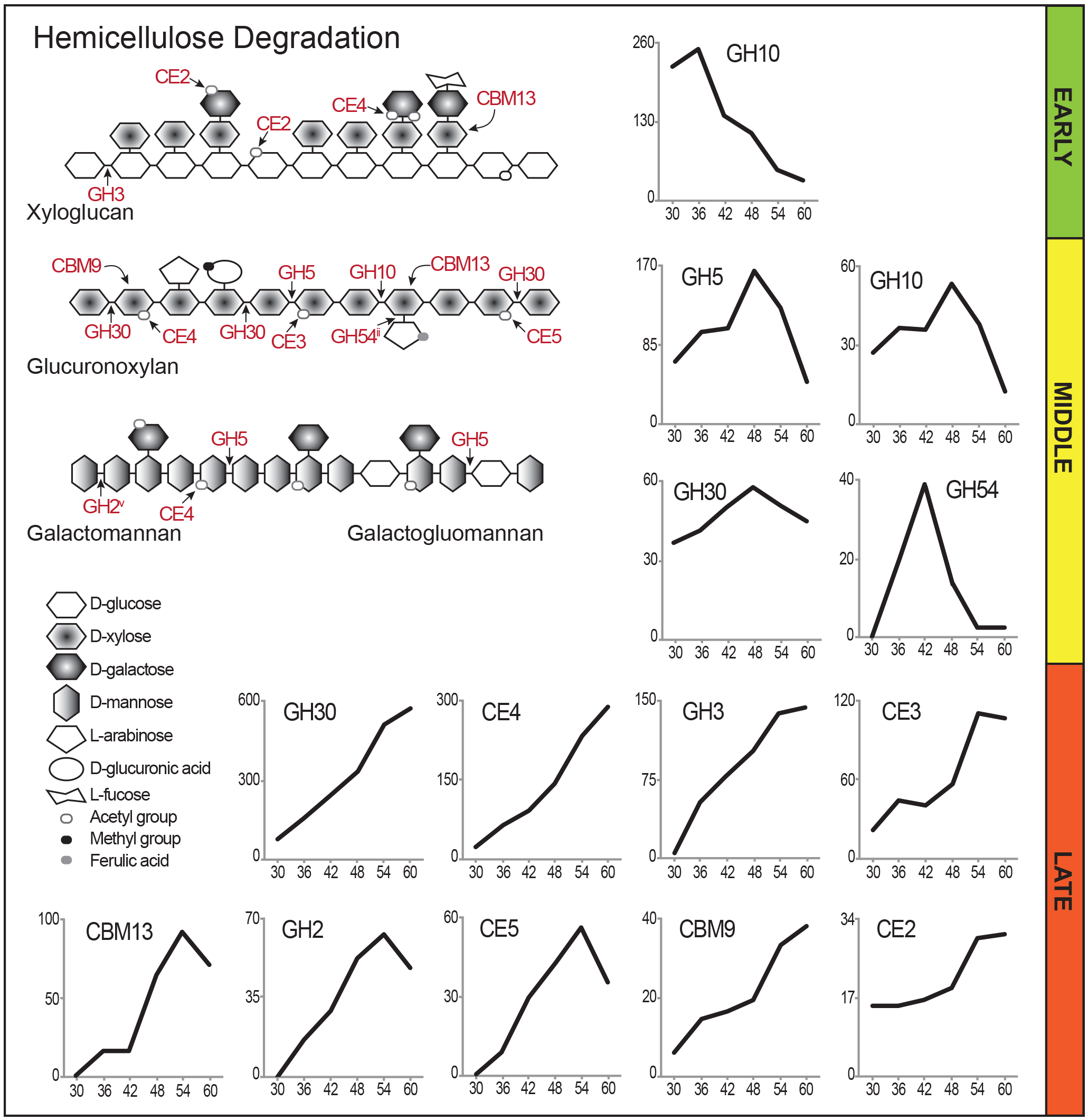

Supplement: S5 Fig — The diagrams show representations of the hemicelluloses xyloglucan, glucuronoxylan and galactomannan/galactoglucomannan and putative sites of activity of proteins from 12 CAZyme families. The vertical axis of each graph shows the total median NRPK values from the multiple location data set for all members of a particular CAZyme family whose highest level of expression occurs at early (30 or 36 hpi), middle (42 or 48 hpi) or late (54 or 60 hpi) stages of infection. The horizontal axis shows the hpi. The number of genes contributing to the data in each graph, the total number of expressed genes in that CAZyme family and the total number of genes in the CAZyme family in the P. parasitica genome are indicated below by the three values separated by slashes. Only genes that had a total NRPK value over the time-course ≥50 have been included. The graphs are arranged in order of decreasing levels of transcript abundance. Some CAZyme families have multiple putative substrate targets: iiproteins that may act on pectins, hemicellulose and glycoproteins; vproteins that may act on hemicellulose and glycoproteins. Early: GH10, 2/4/4. Middle: GH5, 2/20/25; GH10, 1/4/4; GH30a, 1/ 19/21 and GH54, 1/1/1. Late: GH30b, 4/19/21; CE4, 2/2/2; GH3, 1/16/25; CE3, 1/1/1; CBM13, 1/3/4; CE5, 2/4/4; GH2, 1/1/1; CBM9, 1/1/1; CE2, 1/1/1. aThis GH30 also contains a CBM13 domain. bThree of these GH30 proteins also contain a CBM13 domain. (TIF) [file pone.0136899.s005.tif]

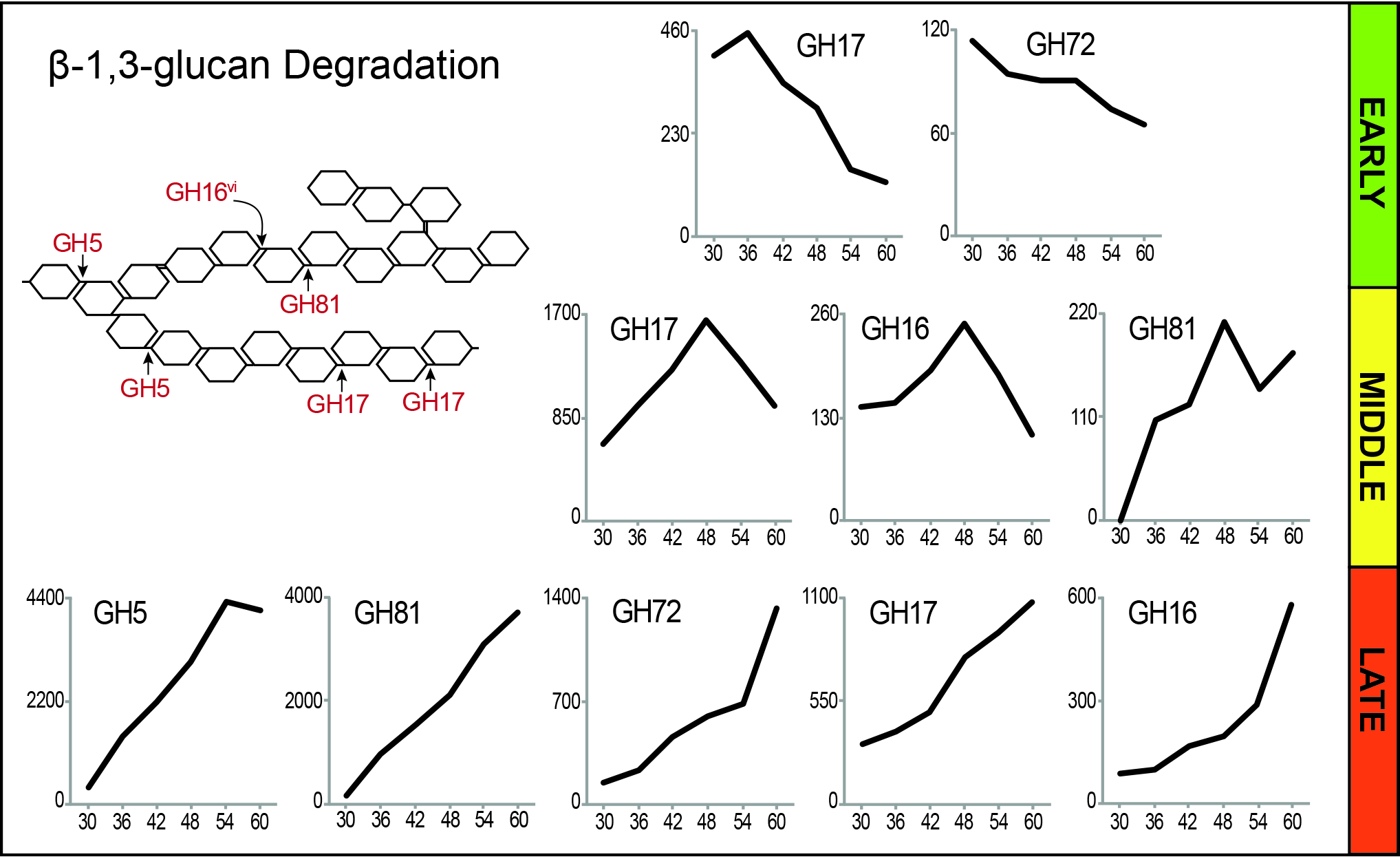

Supplement: S6 Fig — The diagram shows a representation of the structure of β-1,3-glucan and putative sites of activity of proteins from five CAZyme families. The vertical axis of each graph shows the total median NRPK values from the multiple location data set for all members of a particular CAZyme family whose highest level of expression occurs at early (30 or 36 hpi), middle (42 or 48 hpi) or late (54 or 60 hpi) stages of infection. The horizontal axis shows the hpi. The number of genes contributing to the data in each graph, the total number of expressed genes in that CAZyme family and the total number of genes in the CAZyme family in the P. parasitica genome are indicated below by the three values separated by slashes. Only genes that had a total NRPK value over the time-course ≥50 have been included. The graphs are arranged in order of decreasing levels of transcript abundance. Some CAZyme families have multiple putative substrate targets: viproteins that may act on hemicellulose and β-1,3-glucans. Early: GH17a, 5/16/20 and GH72, 2/8/14. Middle: GH17, 2/16/20; GH16, 2/11/16 and GH81, 1/11/16. Late: GH5b, 3/20/25; GH81, 6/11/16; GH72, 6/8/14; GH17, 7/16/20 and GH16, 5/11/16. aTwo GH17 genes also contain a CBM13 domain. bTwo GH5 proteins also contain a CBM43 domain. (TIF) [file pone.0136899.s006.tif]

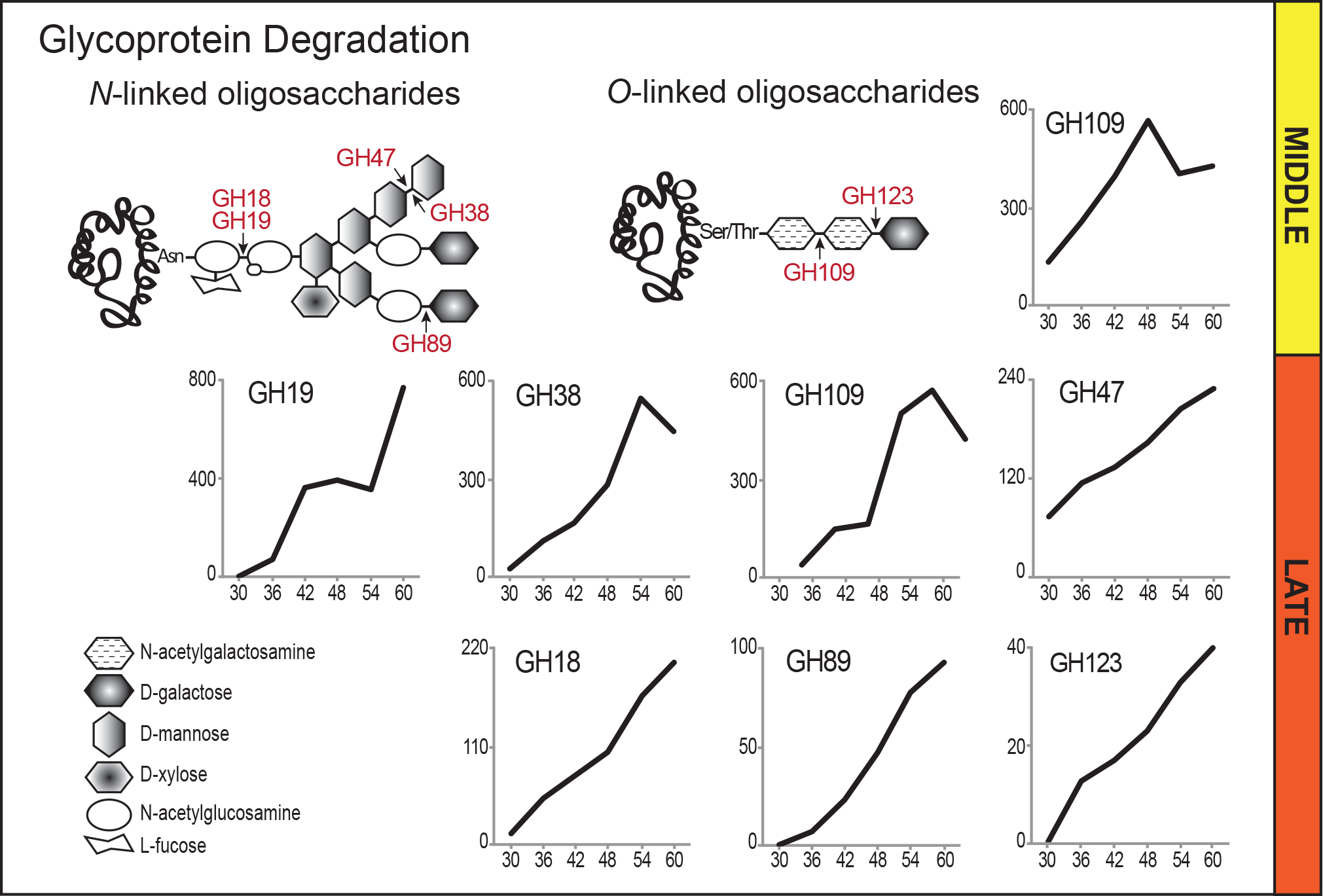

Supplement: S7 Fig — The diagrams show representations of the structures of N- and O-linked glycoproteins and putative sites of activity of enzymes from seven CAZyme families. The vertical axis of each graph shows the total median NRPK values from the multiple location data set for all members of a particular CAZyme family whose highest level of expression occurs at early (30 or 36 hpi), middle (42 or 48 hpi) or late (54 or 60 hpi) stages of infection. The horizontal axis shows the hpi. The number of genes contributing to the data in each graph, the total number of expressed genes in that CAZyme family and the total number of genes in the CAZyme family in the P. parasitica genome are indicated below by the three values separated by slashes. Only genes that had a total NRPK value over the time-course ≥50 have been included. The graphs are arranged in order of decreasing levels of transcript abundance. Middle: GH109, 2/7/7. Late: GH19, 2/2/2; GH38, 1/1/1; GH109, 5/7/7; GH47, 5/5/5; GH18, 2/2/3; GH89, 2/2/2 and GH123, 1/1/1. (TIF) [file pone.0136899.s007.tif]
