# Supplementary material for: RNA-Seq Analysis of the Expression of Genes Encoding Cell Wall Degrading Enzymes during Infection of Lupin (Lupinus angustifolius) by Phytophthora parasitica
Source: PLoS One. 2015 Sep 2;10(9):e0136899. doi: 10.1371/journal.pone.0136899 (PMC4558045; doi:10.1371/journal.pone.0136899)
Supplement: S1 Table — (DOCX) [file pone.0136899.s008.docx]

S1 Table: *P. parasitica* and lupin primers used for qPCR.

| Family/Gene | Accession | Primers (Forward-Reverse: 5’-3’) |
| --- | --- | --- |
| WS041 | CF891677 | CGGTCCCATTGTGCTCTATT-TTGTGTCTTTGTGTGATGCG |
| NIT4A | DQ241759 | TCCAGACTCCAGAAGCAGAG-AAGCACCACACCAAAACTACAA |
| AA8 | PPTG_04148 | TGGTGGTGCTGGTAGTGC-TGCTCCTGAGTTGGTTGATG |
| CBM40 | PPTG_14659 | GGATGCCGAGAGGAACTTG-CCTGAGCGTAATAGTAGGGAAAAC |
| CBM63 | PPTG_12541 | TGGACGGAGCCTTCTACTACC-ACACACTCGCCCACCTTC |
| CE8 | PPTG_05287 | CGCACCGATAACTCAAACC-TGTCACCGTTCCACTTCTG |
| CE8 | PPTG_06239 | GCTGTGGACTTTGTTTTTGGC-TGGAGGCATTGGAGTTGAAC |
| CE8 | PPTG_09705 | GGCTACCAGGACACCGTTC-GAGTTGACACCGCTCTTGC |
| CE8 | PPTG_10388 | TGACGGGCTACCAAGACAC-ACAGACCAAAGACGAAATCCAC |
| CE8 | PPTG_14180 | ACTGTGGGTGAAGGTGCTATC-GTTGGCGGTGTTGTTGTC |
| CE12 | PPTG_16433 | GACCGCTTCAACAAATGGC-TTGCTGGAAAACAACTGCTC |
| GH5 | PPTG_03499 | ACGAGGAGACCGAAACCAC-ACAGCAGGGGCATCAGTG |
| GH5 | PPTG_18765 | TCAAGGTCTCGGGAAGCAG-GTGGCAATGGGGTAATCGTC |
| GH6 | PPTG_00142 | GACCACGGAGACTAACACTGCC-ATACGAGACGACAAGGAGCC |
| GH7 | PPTG_18587 | TGCTAAGAACCGCTACAACG-AAAAACGCTGGACATCAACC |
| GH7 | PPTG_18589 | GACAGCAACTCGGGAACTG-ACGGGTGGGAGGTGTACG |
| GH10 | PPTG_07666 | AGATGCTCGCCGCTTTAG-CGCCGTCGTGATGTAGTC |
| GH10 | PPTG_17240 | CACTGCTCTCGGATTGGAC-TGAACTGCTGCTTTTGCTC |
| GH10 | PPTG_17850 | CAGCGAACAGCAACAACAG-GAGCCGACGATTTTATCCAC |
| GH10 | PPTG_17851 | TGTCCAACCACATTACCAAGG-ACGCCTTCAACCCAAATC |
| GH12 | PPTG_19377 | AAGACTATCCCGACCAAGATG-GGCGACGAACGAGAAGAC |
| GH16 | PPTG_16550 | CAGCAGTCAACCCCTTTCAG-AGATTTTCTTACCACCAGTCCAG |
| GH16 | PPTG_12451 | CTGGCGTGGTCTGTGTGG-TGTTGCTGTGAGGGTCTTTC |
| GH28 | PPTG_15162 | TGAGCAGAAACAACCACG-TGACCAGCAGACCAGAGAC |
| GH28 | PPTG_15164 | GGGACCGCTAAGAACACG-GGAGACGAGACCCTTGAGAC |
| GH28 | PPTG_15171 | GCGCATCAAGACCATCATC-TTACGACGACAGACCAGAATC |
| GH28 | PPTG_15179 | CCGAGAAGACCACAATCAGC-CCTCCGAGCGAACCAATG |
| GH28 | PPTG_17704 | TGGCGGTCACGGTATCTC-CCTCCTTTTTCCTTGCTGTAGTC |
| GH30 | PPTG_14860 | CGGTGGTAAAACAGGCAAAAG-GCAGTGAGCGTGGAGAGTG |
| GH31 | PPTG_07818 | CGGTGGTAAAACAGGCAAAAG-GCAGTGAGCGTGGAGAGTG |
| GH32 | PPTG_09926 | CTCAAACGGCAGCAACTACC-AGCGGCGAACAGCATCAC |
| GH43 | PPTG_15711 | TGGCAAGGCATTTACCAGAC-GCGACTCAAGCACAACGG |
| GH43 | PPTG_17407 | CGTTCTAAGTCTGGCGTAATGG-TTAGTTTTCCTGGGTCGTTTG |
| GH53 | PPTG_19167 | TCACGCTGAGTGCTTCCTAC-CTGCCGAGAACCCTTACG |
| GH105 | PPTG_07904 | CTCGGTATGCTCAAAGACAAAG-GGCTCGCCAAAATGAAGG |
| PL1 | PPTG_12896 | CCACGCCGCTAATAACTACTTC-TTTCTTCGCCGCATCTTC |
| PL1 | PPTG_17499 | GTTTGAGTCGGTGAACGG-GCAGCGGGAGTAGGGTC |
| PL3 | PPTG_04818 | AGGAATCACGACCATCACTG-TAGAACCCGCCACTGACAAC |
| PL4 | PPTG_05072 | AAGGAGCAGGTCGGTAAGG-TCACGGCAGCACTCATTG |
| PL4 | PPTG_05074 | CTACGCCATTCCCAAGACTG-GCCGACAACCATTTTCCAAG |
| PL4 | PPTG_05103 | CGGCAAGGACGACAACAG-CCCCCACAGACTCAGGAAG |
