# Supplementary material for: RNA-Seq Analysis of the Expression of Genes Encoding Cell Wall Degrading Enzymes during Infection of Lupin (Lupinus angustifolius) by Phytophthora parasitica
Source: PLoS One. 2015 Sep 2;10(9):e0136899. doi: 10.1371/journal.pone.0136899 (PMC4558045; doi:10.1371/journal.pone.0136899)
Supplement: S2 Table — (DOCX) [file pone.0136899.s009.docx]

S2 Table. Data for the total number of reads mapped per sample, the lowest quality score for each base across all reads as determined by FastQC, and the number of reads mapped to unique and multiple locations in the *P. parasitica* genome.

| **Sample** | **Total number of reads per sample** | **Lowest quality score across all reads** | **Number of reads mapping to unique *P. parasitica* locations** | **Number of reads mapping to multiple *P. parasitica* locations** |
| --- | --- | --- | --- | --- |
| 0 h | 61,202,816 | 31 | 5,005 | 8,731 |
| 0 h | 58,628,588 | 31 | 4,913 | 8,363 |
| 0 h | 58,789,211 | 31 | 5,095 | 8,960 |
| 12 h | 60,519,488 | 31 | 6,089 | 9,347 |
| 12 h | 62,640,212 | 31 | 5,645 | 8,499 |
| 12 h | 59,760,424 | 31 | 5,132 | 7,653 |
| 18 h | 63,838,110 | 31 | 6,243 | 9,010 |
| 18 h | 63,642,384 | 30 | 7,195 | 10,141 |
| 18 h | 61,349,687 | 30 | 5,071 | 7,724 |
| 24 h | 60,551,957 | 31 | 83,304 | 89,424 |
| 24 h | 59,773,029 | 30 | 23,588 | 27,141 |
| 24 h | 63,941,124 | 30 | 11,142 | 14,572 |
| 30 h | 63,051,370 | 30 | 228,492 | 241,597 |
| 30 h | 62,239,393 | 30 | 137,079 | 146,128 |
| 30 h | 62,760,749 | 30 | 82,858 | 89,686 |
| 36 h | 60,765,498 | 30 | 405,936 | 426,117 |
| 36 h | 62,527,298 | 30 | 484,957 | 510,316 |
| 36 h | 64,663,110 | 30 | 733,938 | 772,185 |
| 42 h | 64,884,720 | 30 | 3,623,492 | 3,786,615 |
| 42 h | 64,675,943 | 30 | 2,088,074 | 2,182,184 |
| 42 h | 65,668,183 | 30 | 2,508,480 | 2,623,026 |
| 48 h | 60,407,739 | 31 | 5,737,603 | 6,018,593 |
| 48 h | 60,465,332 | 31 | 4,033,022 | 4,236,421 |
| 48 h | 60,253,447 | 31 | 7,645,002 | 8,027,208 |
| 54 h | 60,363,823 | 31 | 12,893,233 | 13,445,398 |
| 54 h | 65,955,334 | 31 | 9,966,320 | 10,452,928 |
| 54 h | 65,664,529 | 31 | 9,073,961 | 9,542,374 |
| 60 h | 64,464,862 | 31 | 11,911,182 | 12,412,470 |
| 60 h | 64,738,698 | 31 | 17,403,585 | 18,173,591 |
| 60 h | 62,461,038 | 31 | 14,287,966 | 14,954,163 |
